# Are healthy ageing trajectories suitable to identify rehabilitation needs of the ageing population? An exploratory study using ATHLOS cohort data

**DOI:** 10.1371/journal.pone.0303865

**Published:** 2024-07-09

**Authors:** Carla Sabariego, Jsabel Hodel, Vanessa Seijas, Angel Rodriguez-Laso, Jerome Bickenbach, Cristina Ehrmann

**Affiliations:** 1 Swiss Paraplegic Research, Nottwil, Switzerland; 2 Faculty of Health Sciences and Medicine, University of Lucerne, Lucerne, Switzerland; 3 Center for Rehabilitation in Global Health Systems, University of Lucerne, Lucerne, Switzerland; 4 CIBER de Fragilidad y Envejecimiento Saludable (CIBERFES), Instituto de Salud Carlos III, Madrid, Spain; Royal College of Surgeons in Ireland, IRELAND

## Abstract

**Background:**

The ageing process is highly heterogeneous leading to diverse ageing trajectories. Such trajectories have been modelled to study trends and determinants of ageing and could potentially be used to inform the planning of rehabilitation services at population level. The objective of this paper was to explore whether healthy ageing trajectories are suitable to identify targets for rehabilitation interventions for the ageing population.

**Methods:**

Data from the Ageing Trajectories of Health: Longitudinal Opportunities and Synergies (ATHLOS) harmonized cohort and the English Longitudinal Study of Ageing (ELSA), which is included in ATHLOS, were used. Growth mixture models were implemented to replicate healthy ageing trajectories modelled elsewhere as ’high stable’, ’low stable’, and ’rapid decline’. Regularized partial correlation networks were used to estimate expected-influence and bridge expected-influence centrality measures.

**Results:**

Healthy ageing trajectories modelled with ATHLOS (N = 131116) and ELSA (N = 14904) were consistent with previous work. At the first individual wave, prevalence of problems of respondents in the ’high stable’ and ’rapid decline’ trajectories were comparable; at the last individual wave, prevalence remained similar for the ’high stable’ but increased substantially in all domains for the ’rapid decline’ trajectory. Expected-influence centrality measures provided different results than prevalence estimates. For instance, in the first individual wave mobility and carrying, moving, and handling objects had highest impact on overall functioning for the ’rapid decline’ and ’high stable’ trajectories, while the most prevalent functioning problems concerned cognition, pain, as well as energy and drive functions.

**Conclusions:**

Our study stressed the relevance of healthy ageing trajectories for identifying functioning domains and categories that need to be targeted by rehabilitation services in a heterogeneous ageing population. The use of such trajectories at country level has the potential to make a meaningful contribution to the planning and delivery of rehabilitation interventions through health systems and to informed policy making.

## Introduction

The global population is rapidly ageing. Global projections reckon that by 2050 the world will have approximately 1.5 billion of older persons, and that one in six persons will be 65+ [1]. The 2021–2030 United Nations (UN) Decade of Healthy Ageing (henceforth referred to as the Decade) [2] calls therefore for global commitment to respond to the challenges posed by this trend. Healthy ageing is a complex concept defined not as an outcome but as a process of "developing and maintaining the functional ability that enables well-being in older age" [3], whereas functional ability is defined as “the health-related attributes that enable people to be and to do what they have reason to value” and is the outcome of the interplay between a person´s intrinsic capacity (his or her health state including physical and mental capabilities) and his or her environment, including physical, attitudinal, social and political dimensions. Leading the Decade, the World Health Organization (WHO) has defined that strengthening health systems to provide "person-centred integrated care and primary health services responsive to older people" is a key area for action to achieve healthy ageing [3].

Rehabilitation is a person-centered health strategy that aims to optimize the functioning of persons with health conditions, considering the built, social and political context of their lives [4]. The concept of functioning has been defined in the International Classification of Functioning, Disability and Health (ICF) as an umbrella term encompassing body functions, such as sleep or memory functions, activities, such as walking or self-care, and participation, such as working or attending family celebrations [5]. Moreover, functioning is described as the outcome of the interaction between a person with a health condition and the context where the person lives, including how the place of living is built, the attitudes of society, friends and family, the availability of social support, social and political regulations, among others. The concept of functioning, which is key for rehabilitation, is in our appraisal very similar to the WHO definition of functional ability used in the Healthy Ageing agenda.

Recent Global Burden of Disease (GBD) Study estimates showed that around 2.4 billion persons living with mostly chronic health conditions can potentially benefit from rehabilitation at some point in life [6]. From these, a large proportion have diseases that are associated to the ageing process, such as musculoskeletal conditions or the loss of sensory functions like hearing and vision loss [6]. Indeed, rehabilitation in older ages focuses both on limitations associated with age-related declines in intrinsic capacity as well as on problems linked to chronic or incurable health conditions, mostly non-communicable diseases (NCDs). Rehabilitation can therefore meaningfully contribute to achieving the goals of the Decade, and, as argued elsewhere, a failure to integrate rehabilitation into the healthy ageing agenda would be a lost opportunity [7]. Nevertheless, rehabilitation has not yet been integrated to its full potential in the global healthy ageing agenda.

WHO acknowledges that people age in different ways so that the ageing process is highly heterogeneous leading to diverse ageing trajectories [3]. WHO described in the World Report on Ageing and Health (WRA) launched in 2015 that some people may age with minor limitations while others may develop several, relatively minor health problems, for instance in mobility, vision or hearing, or memory, that taken together considerably lower their functional ability [3]. Others may live most of their lives with a serious and progressive health problem leading to a continuous decline of their intrinsic capacity and functional ability. These diverse trajectories have been modelled in a European Commission (EC) project called Ageing Trajectories of Health: Longitudinal Opportunities and Synergies (ATHLOS), in which data from 16 international cohorts was harmonized to create a common scale of intrinsic capacity and functional ability for 343915 individuals [8]. When longitudinally modelled over a 20 years period, three ageing trajectories were observed: ’high stable’, ’low stable’ and ’rapid decline’ [9]. The first two trajectories start with different levels of intrinsic capacity and functional ability but have both a slow and minor decline over time while the latter is characterized by a continuous and fast decline. These trajectories have been broadly used in research, for instance to investigate the role of multimorbidity on ageing [10], and can be relevant for planning and delivering health services through health systems.

An open question is whether healthy ageing trajectories can be used to inform the planning of rehabilitation services at population level. Modelling intrinsic capacity and functional ability for populations over time might prove suitable to identify trajectory-specific targets for rehabilitation interventions as well as critical service delivery time points and windows of opportunity. However, to the best of our knowledge this has not been done so far. The objective of this paper is therefore to explore whether healthy ageing trajectories are suitable to identify targets for rehabilitation interventions for the ageing population. For doing so, we use in this explorative study the healthy ageing scale, called healthy ageing index (HAI), created in ATHLOS [8], as well as the ATHLOS harmonized cohort data [10]. To identify modifiable targets for rehabilitation interventions we estimate the prevalence of functioning problems as well as the importance of each functioning problem in the multivariate structural dependence among all and domain specific functioning problems using regularized partial networks and centrality measures. The higher the value of the centrality measures, the more likely is the functioning problem a candidate for intervention. In summary, we aim to answer two questions about persons with regard to the identified three distinct ageing trajectories: a) What is the prevalence of functioning problems, and; b) Which functioning categories have a central impact on overall functioning and on specific functioning domains.

## Materials and methods

### Data and sample

The main sample used in our data analysis is the ATHLOS harmonized cohort [9], which includes data from 16 international cohorts. Two criteria were used to select from the ATHLOS harmonized cohort for our study: 1) included cohort studies needed to have at least three waves of data collection and 2) included persons of the selected cohort studies needed to have participated in at least two time points, in order to establish their trajectories. Using this criteria, we used data from the Australian Longitudinal Study of Ageing (ALSA) waves 1 to 11 [11], the English Longitudinal Study of Ageing (ELSA) waves 1 to 7 [12], the Study on Cardiovascular Health, Nutrition and Frailty in Older Adults in Spain (ENRICA) waves 1 to 3 [13–15], the Health and Retirement Study (HRS) waves 1 to 11 [16, 17], the Korean Longitudinal Study on Health and Ageing (KLOSA) waves 1 to 4 [18], the Mexican Health and Ageing Study (MHAS) waves 1 to 3 [19, 20], and the Survey of Health, Ageing and Retirement in Europe (SHARE) waves 1 to 5 [21–27]. Approval for using these data has been obtain from the ATHLOS Intellectual Property and Dissemination Board. The difference between first time point and last time point of assessment of respondents had a median of 2 waves, with a minimum of one wave and a maximum of 10 waves of difference.

For planning of rehabilitation services, it is important to identify longitudinal trends considering the context of a country or region, what is not possible with the rather heterogeneous ATHLOS harmonized cohort. To get a better overview of how rehabilitation intervention targets change over time in a specific setting, we additionally used data from ELSA, which is one of the studies included in the ATHLOS harmonized cohort, as an exemplary survey to conduct the same analyses with the possibility of examining changes over time over its seven assessment waves.

### Variables

For replicating the healthy ageing trajectories published elsewhere [10], the HAI score was used [8]. The HAI (the higher, the better) is a metric score developed using the two-parameter logistic Item Response Theory model [28] and 41 items addressing intrinsic capacity and functional ability, referred to in our publication as functioning items. These items are understood as the specific ATHLOS questions/assessments and corresponding dichotomized response options (having a problem in functioning/ having no problem in functioning) that were used to assess the functioning information. They include both self-reported items and measurements using performance tests. ICF categories were used to classify the 41 functioning items. The categorized/classified functioning items are used as functioning variables in the statistical analysis of this study and can be summarized according to ICF domains, which are practical and meaningful category groupings corresponding to either ICF chapters (like mobility) or second level categories (like seeing or hearing functions) (S1 Table).

Descriptive statistics were used to characterize the sample regarding sex, age, education, wealth, smoking and drinking patterns, and multimorbidity. For the sake of consistency, we took over a previously used definition of multimorbidity [10], namely having two of more health conditions.

### Statistical analysis

#### Replication of healthy ageing trajectory classes

To ensure consistency with previous work, we first replicated the estimation of the ageing trajectories modelled using the ATHLOS harmonized cohort [10]. We used the baseline growth mixture model (GMM) [10]. This model allows us to accommodate heterogeneity within a population by assuming and identifying unobserved subgroups (i.e. latent classes) in a sample of individual healthy ageing trajectories and to describe longitudinal change with respect to each latent class. While for ATHLOS data the GMM was performed over 11 time points, covering a period up to 22 years, for the ELSA study, which is part of the ATHLOS harmonized cohort, 7 time points were considered. As in the previous study, we modelled the change in the trajectories to be linear. The decision for the optimal number of latent trajectory classes was based on the following criteria: 1) lowest Akaike, Bayesian, and sample-size adjusted Bayesian information criteria (AIC, BIC and SABIC, respectively) [29], 2) highest entropy value [30], 3) p-value < 0.001 for the Vuong-Lo-Mendell-Rubin likelihood ratio test (LMR LR) [31], for the Lo-Mendell- Rubin adjusted likelihood ratio test (aLMR LR) [31], and for the parametric bootstrapped likelihood ratio test (BLRT) [32], 4) no class size < 1% of the study sample, and 5) average posterior probabilities for each latent class > 0.70 [30]. Missing values were assumed to be missing at random and imputed using the full information maximum likelihood technique. GMM was conducted using MPlus version 8.2 [33].

#### Prevalence of and associations between functioning problems

For each trajectory class, overall prevalence of functioning problems was calculated. For the ATHLOS data, we draw two samples for each trajectory: the respondents first and last waves of individual assessment of functioning variables used to build the HAI, given that around 50% of respondents included in this study had HAI scores only for two waves (53821 respondents out of 131116). For ELSA, which is one of the studies included in the ATHLOS harmonized cohort, we used data from all available seven waves. Since all available responses on functioning variables with less than 20% missing observations were included in each analysis, each sample may have a different number of variables [34]. To deal with missing observations, a random forest imputation technique was used implementing the R package Missforest (version 1.4) [35].

**Fig 1:**
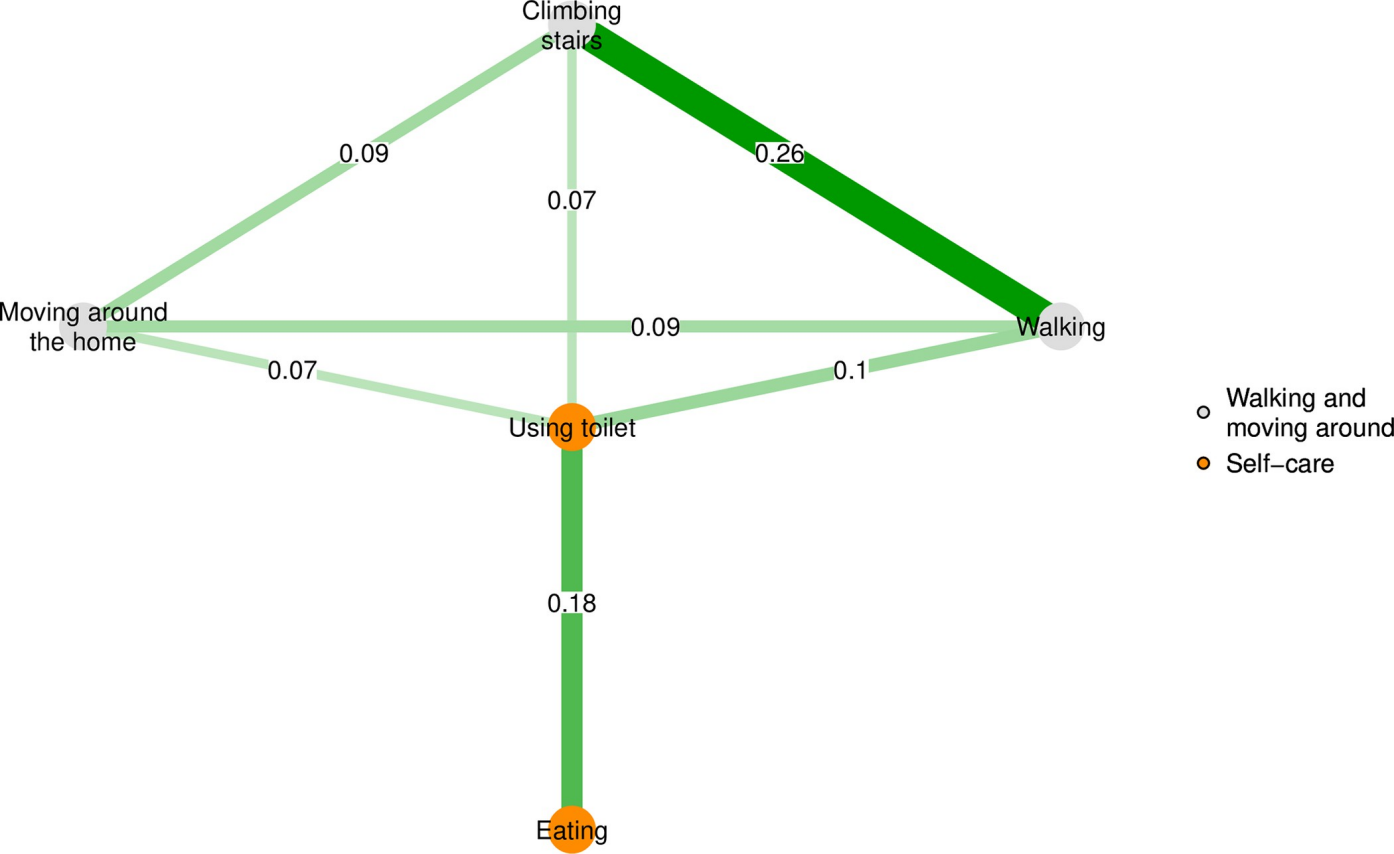
Example of a regularized partial correlation network (RPCN) returned via the graphical least absolute shrinkage and selection operator (LASSO), which is depicting associations between pairs of functioning variables. The node colors indicate ICF domains.

To study associations a regularized partial correlation network (RPCN) was estimated for each sample. RPCNs consist of nodes and edges. While nodes represent the functioning variables of each sample, the edges represent conditional associations among the variables, or more specifically, partial correlations between pairs of variables controlling for the influence of all remaining variables. The partial correlation between two nodes is called edge weight. To limit the number of spurious edge weights between pairs of nodes, the graphical least absolute shrinkage and selection operator (LASSO) regularization technique was used. The graphical LASSO regularization was tuned using the Extended BIC (EBIC) and the EBIC hyperparameter γ (gamma) for controlling the trade-off between the removal of true edges and the inclusion of false-positive edges [36]. For obtaining sparse network structures, we selected a conservative value of γ = 0.5. The R-packages bootnet (version 1.5) [36] and qgraph [37] (version 1.9.2) were used for the RPCN estimations and visualizations, respectively. Fig 1 shows an example visualization of a RPCN with 5 selected functioning variables as nodes. The edge weights indicate the estimated partial correlation between the variables. The larger the correlation, the thicker is the edge in the graph.

#### Functioning variables with central impact on overall and domain-specific functioning

The expected-influence and one-step bridge expected-influence centrality measures of each node were estimated to describe the connectivity of nodes within the RPCNs [38, 39]. While the expected-influence of a node is the sum of the edge weights linking the node with the remaining nodes, the one-step bridge expected-influence is the sum of the edge weights connecting a given node to all nodes in other ’communities’, i.e., groups of variables defined a priori. In this study, communities correspond to ICF domains. A high expected-influence value of a node indicates a high connectivity to all other nodes in the RPCN, i.e. an intervention targeting this functioning aspect has the potential to impact the problems in the remaining functioning variables (conditional on evidence about causal relationships). A high bridge expected-influence of a node within a specific ICF domain indicates high connectivity, and thus, influence to the nodes in all other remaining ICF domains in the RPCN, i.e. an intervention in this functioning variable (target) is likely to affect the functioning variables from other ICF domains. In Fig 1, the value of the expected-influence associated to the node ’climbing stairs’ is 0.42 (sum of edge weights connecting ’climbing stairs’ with the nodes ’moving around the home’, ’walking’ and ’using toilet’), while the value of the one-step bridge expected-influence is 0.07 (edge weight between ’climbing stairs’, a variable of the ’walking and moving around’ ICF domain, and ’using toilet’, a variable of the ’self-care’ ICF domain). Both expected-influence estimates were derived using the R-package bootnet. To check if network results can be replicated, the accuracy of edge weights and the stability of expected-influence estimates were examined. The accuracy of edge weights was assessed by employing a non-parametric bootstrap approach to calculate the 95% confidence intervals (95% CI) for the edges by sampling the data 10’000 times (with replacement). Greater accuracy is indicated by smaller confidence intervals. The stability of expected-influence estimates was checked with the correlation stability coefficient calculated between the original expected-influence estimates (based on the full data) and the expected-influence estimates obtained from subset of data representing different percentages of the full data (case dropping bootstrapped from the full data by sampling the data 10’000 times). Good stability is indicated by a correlation stability coefficient greater than 0.5 [36].

## Results

### Replication of healthy ageing trajectory classes

**ATHLOS.** The sample used to replicate the ATHLOS trajectory classes estimated elsewhere included 131’116 individuals At the first individual wave, participants were on average 62.1 years old (SD = 10.7), 56.5% were female and 24.7% had at least one comorbidity (Table 1). Moreover, 81.1% of the respondents were alive at the individual last wave of assessment of the corresponding survey, i.e., potentially able to participate in the next wave. Table 2 presents the results of the GMMs for ATHLOS, where two to five trajectory classes were considered. Although the AIC, BIC and SABIC decrease as the number of classes increase, the four- and five-class models had an average posterior probability lower than the threshold of 0.7 and some identified classes comprised only 1% of the sample. Moreover, the three-class model was favored by the highest entropy. This study confirm therefore the three-class model of the reference study [10] as the optimal solution.

**Table 1:** ATHLOS sample characteristics at the first individual wave including the living status at the last individual wave. The total number of non-missing cases is used to calculate the percentage.

	ALSA (N = 1870)	ELSA (N = 14904)	ENRICA (N = 2516)	HRS (N = 34141)	KLOSA (N = 8928)	MHAS (N = 13880)	SHARE (N = 54877)	ATHLOS Total (N = 131116)
**Age (SD)**	79.9 (6.6)	61.3 (10.2)	68.7 (6.4)	59.9 (11.0)	61.6 (10.9)	60.6 (10.4)	63.4 (10.1)	62.1 (10.7)
**Sex, n (%)**								
Female	939 (50.2)	8217 (55.1)	1336 (53.1)	19386 (56.8)	5054 (56.6)	7898 (57.7)	31117 (56.7)	73947 (56.5)
Male	931 (49.8)	6687 (44.9)	1180 (46.9)	14755 (43.2)	3874 (43.4)	5789 (42.3)	23760 (43.3)	56976 (43.5)
**Education, n (%)**								
Less than primary education	16 (1.0)	4960 (36.4)	418 (16.6)	-	1579 (17.7)	3313 (24.0)	1947 (3.6)	12233 (9.5)
Primary education	556 (35.2)	-	953 (37.9)	8885 (26.0)	2499 (28.0)	7396 (53.6)	11253 (20.8)	31542 (24.5)
Secondary education	924(58.4)	6385 (46.9)	614 (24.4)	18873 (55.3)	3897 (43.7)	2297 (16.6)	29863 (55.3)	62853 (48.9)
Tertiary education	85(5.4)	2281 (16.7)	531 (21.1)	6372 (18.7)	951 (10.7)	802 (5.8)	10950 (20.3)	21972 (17.1)
**Wealth, n (%)**								
Quintile 1 (lowest)	610 (35.0)	2262 (17.7)	-	6620 (19.4)	1634 (19.8)	2617 (21.3)	10280 (18.8)	24023 (19.4)
Quintile 2	786 (45.1)	2308 (18.1)	-	6751 (19.8)	1676 (20.3)	2285 (18.6)	10458 (19.2)	24264 (19.6)
Quintile 3	-	2498 (19.6)	-	6923 (20.3)	2144 (26.0)	2469 (20.1)	10814 (19.8)	24484 (20.1)
Quintile 4	-	2739 (21.5)	-	6885 (20.2)	1282 (15.6)	2466 (20.0)	11473 (21.0)	24845 (20.1)
Quintile 5 (highest)	345 (19.8)	2952 (23.1)	-	6964 (20.4)	1502 (18.2)	2466 (20.0)	11552 (21.2)	25779 (20.8)
**Smoking, n (%)**								
Never smoked	892(48.5)	5675 (38.9)	1346 (53.5)	14248 (42.0)	6354 (71.2)	7982 (57.5)	29133 (53.6)	65630 (50.5)
Ever smoked	948 (51.5)	8884 (61.0)	1170 (46.5)	19710 (58.0)	2573 (28.8)	5892 (42.5)	25234 (46.4)	64411 (49.5)
**Drinking, n (%)**								
Never	673 (36.3)	1418 (10.3)	893 (36.0)	17453 (51.2)	312 (9.2)	2386 (56.1)	16900 (30.9)	40035 (34.9)
Rare	491 (26.5)	8041 (58.3)	207 (8.3)	9985 (29.3)	1787 (52.5)	1221 (28.7)	22404 (40.9)	44136 (38.5)
Often	688 (37.1)	4325 (31.4)	1380 (55.6)	6651 (19.5)	1307 (38.4)	647 (15.2)	15422 (28.2)	30420 (26.5)
**Multimorbidity, n (%)**								
Presence	720 (38.5)	3825 (25.7)	596 (23.7)	9549 (28.0)	1490 (16.7)	2902 (21.5)	13214 (24.1)	32296 (24.7)
Absence	1150 (61.5)	11073 (74.3)	1920 (76.3)	24590 (72.0)	7438 (83.3)	10599 (78.5)	41607 (75.9)	98377 (75.3)
**Living status at the last individual wave, n (%)**								
Alive	134 (7.2)	13169 (88.4)	1821 (72.4)	19693 (62.0)	7104 (79.6)	9492 (68.4)	53014 (96.7)	104427 (81.1)
Dropout due to the death	985 (52.7)	1735 (11.6)	50 (2.0)	10749 (33.8)	539 (6.0)	2635 (18.0)	1811 (3.3)	18504 (14.4)
Dropout from unknown reason	751 (40.1)	-	645 (25.6)	1357 (4.2)	1285 (14.4)	1753 (12.6)	-	5791 (5.5)

N = number, SD = standard deviation.

**Table 2:** Model fit information of linear growth mixture models in ATHLOS and ELSA samples. ELSA is one of the studies included in the ATHLOS harmonized cohort and was used to identify longitudinal trends considering the context of a country, what is not possible with the rather heterogeneous ATHLOS harmonized cohort.

	ATHLOS (N = 131116)				ELSA (N = 14904)			
Number of classes	2 classes	3 classes	4 classes	5 classes	2 classes	3 classes	4 classes	5 classes
Number of parameters	9	22	25	28	15	18	21	24
AIC	3379003	3374560	3372891	3371255	441393	440742	440487	440350
BIC	3379091	3374775	3373135	3371529	441507	440879	440647	440533
SABIC	3379062	3374705	3373056	3371440	441459	440822	440580	440456
LMR LR p-value	<0.001	<0.001	<0.001	<0.001	<0.001	<0.001	<0.001	<0.001
aLMR LR p-value	<0.001	<0.001	<0.001	<0.001	<0.001	<0.001	<0.001	<0.001
BLRT p-value	<0.001	<0.001	<0.001	<0.001	<0.001	<0.001	<0.001	<0.001
Entropy	0.61	0.69	0.58	0.65	0.71	0.76	0.77	0.70
Class size, N(%)								
Class 1	98605	2176	33553	71250	4751	499	9802	505
	(75%)	(2%)	(26%)	(55%)	(32%)	(3%)	(66%)	(3%)
Class 2	32511	29175	2186	1789	10153	4276	131	135
	(25%)	(22%)	(1%)	(1%)	(68%)	(29%)	(1%)	(1%)
Class 3		99765	89288	9659		10129	4318	2453
		(76%)	(68%)	(7%)		(68%)s	(29%)	(17%)
Class 4			6089	2008			653	3421
			(5%)	(2%)			(4%)	(23%)
Class 5				46410				8390
				(35%)				(56%)
Average latent class probabilities								
Class 1	0.81	0.71	0.73	0.84	0.88	0.92	0.92	0.69
Class 2	0.90	0.77	0.68	0.70	0.94	0.75	0.76	0.75
Class 3		0.89	0.80	0.73		0.84	0.83	0.78
Class 4			0.64	0.68			0.70	0.64
Class 5				0.69				0.89

AIC = Akaike information criteria, BIC = Bayesian information criteria, SABIC = sample-size adjusted Bayesian information criteria, LMR LR = Vuong-Lo-Mendell-Rubin likelihood ratio test, aLMR LR = adjusted Lo-Mendell-Rubin likelihood ratio test, BLRT = bootstrapped likelihood ratio test.

**ELSA.** ELSA is one of the studies included in the ATHLOS harmonized cohort and was exemplarily used to identify longitudinal trends in a specific country. Of 14904 ELSA participants, 55.1% were female. The mean age was 61.3 years (SD = 10.2) and 25.7% had at least one comorbidity (Table 1). Table 2 shows that from two to five trajectory classes, the three-class solution is supported by the sample size and the average posterior probability of the identified classes.

**Fig 2:**
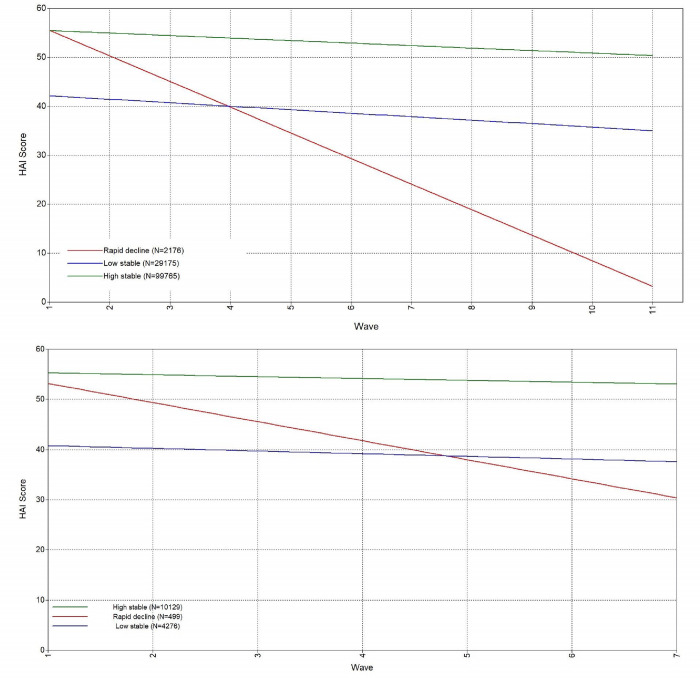
Identified class-specific mean healthy ageing trajectories for ATHLOS (upper figure) and ELSA, which is one of the studies included in the ATHLOS harmonized cohort (bottom figure).

Fig 2 shows the patterns of healthy ageing trajectories that resulted from the three-class model for both samples. We have used the same labels for the trajectory classes as defined by Nguyen et al [10]. Table 3 shows the trajectory-specific sample characteristics of people at the first individuals’ wave. Participants in the ’rapid decline’ trajectory were older than those in the other classes.

**Table 3:** Healthy ageing trajectory classes sample characteristics at baseline for ATHLOS and ELSA. ELSA is one of the studies included in the ATHLOS harmonized cohort and was used to identify longitudinal trends considering the context of a country, what is not possible with the rather heterogeneous ATHLOS harmonized cohort. The total number of non-missing cases is used to calculate the percentage.

	**ATHLOS**				**ELSA**			
	Rapid decline (N = 2176)	Low stable (N = 29175)	High stable (N = 99765)	P-value	Rapid decline (N = 499)	Low stable (N = 4276)	High stable (N = 10129)	P-value
**Sex, n (%)**								
Female	1189 (54.6)	19423 (66.7)	53335 (53.5)	<0.001	262 (52.5)	2667 (62.7)	5288 (52.2)	<0.001
Male	987 (45.4)	9714 (33.3)	46275 (46.5)		237 (47.5)	1609 (37.6)	4841 (47.8)	
**Age (SD)**	71.2 (10.4)	68.8(11.7)	60.1 (9.51)	<0.001	68.6 (10.6)	66.9(10.9)	58.6 (8.6)	<0.001
**Education, n (%)**								
Less than primary education	222 (10.4)	5282 (18.6)	6729 (6.9)	<0.001	222 (49.0)	2249 (58.2)	2489 (26.7)	<0.001
Primary education	714 (33.4)	10393 (36.5)	20435 (20.9)		-	-	-	
Secondary education	956 (44.7)	10773 (37.8)	51124 (52.2)		179 (39.5)	1365 (35.3)	4841 (52.0)	
Tertiary education	246 (11.5)	2020 (7.1)	19706 (20.1)		52 (11.5)	248 (6.4)	1981 (21.3)	
**Wealth, n (%)**								
Quintile 1 (lowest)	481 (23.0)	8587 (31.6)	14955 (15.8)	<0.001	116 (25.3)	1068 (27.0)	1078 (12.9)	<0.001
Quintile 2	453 (21.6)	6901 (25.4)	16910 (17.9)		100 (21.8)	1048 (26.5)	1160 (13.9)	
Quintile 3	444 (21.2)	5146 (18.9)	19258 (20.4)		86 (18.7)	861 (21.8)	1551 (18.6)	
Quintile 4	376 (18.0)	3725 (13.7)	20744 (22.0)		75 (16.3)	604 (15.3)	2060 (24.7)	
Quintile 5 (highest)	340 (16.2)	2846 (10.5)	22593 (23.9)		82 (17.9)	371 (9.4)	2499 (29.9)	
**Smoking, n (%)**								
Never smoked	1143 (52.9)	14810 (51.2)	49677 (50.2)	0.001	173 (35.5)	1360 (32.4)	4142 (41.9)	<0.001
Ever smoked	1015 (47.1)	14102 (48.8)	49294 (49.8)		315 (64.5)	2834 (67.6)	5735 (58.0)	
**Drinking, n (%)**								
Never	837 (43.8)	12440 (50.6)	26758 (30.4)	<0.001	47 (9.9)	765 (19.0)	606 (6.5)	<0.001
Rare	672 (35.2)	7873 (32.0)	35591 (40.4)		284 (59.9)	2369 (58.8)	5388 (58.0)	
Often	402 (21.0)	4279 (17.4)	25739 (29.2)		143 (30.2)	893 (22.2)	3289 (35.4)	
**Multimorbidity, n (%)**								
Presence	566 (26.1)	14671 (50.4)	17059 (17.2)	<0.001	151 (31.3)	2256 (52.8)	1413 (14.0)	<0.001
Absence	1602 (73.9)	14429 (49.6)	82346 (82.8)		343 (68.7)	2016 (47.2)	8714 (86.0)	
**Health ageing score at baseline (SD)**	54.8 (6.7s)	39.8 (6.2)	54.6 (6.9)	<0.001	52.9 (5.5)	40.0 (5.7)	55.0 (5.9)	<0.001

N = number, SD = standard deviation; P-values from χ^2^ tests for the comparison of health ageing trajectory classes groups.

**Fig 3:**
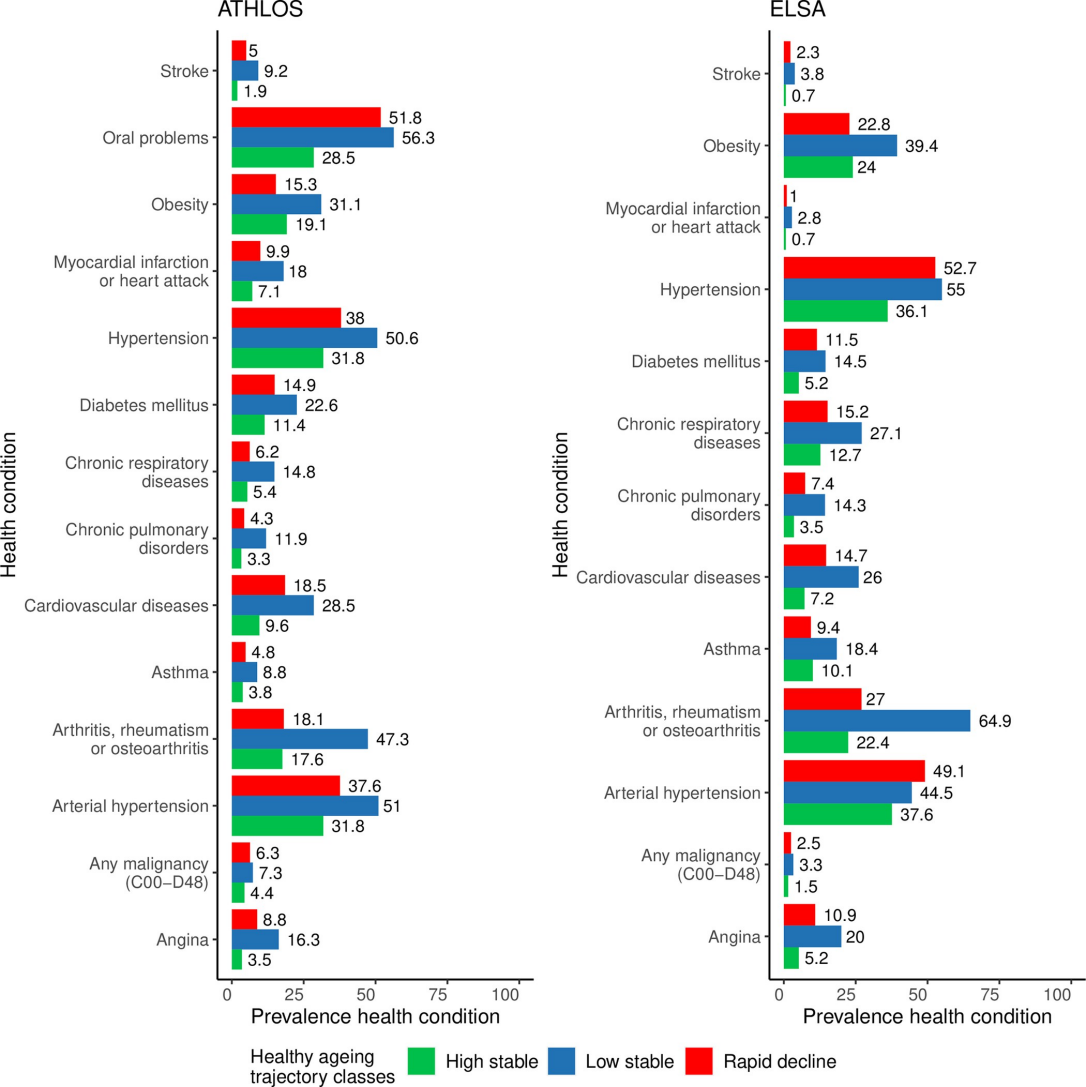
Prevalence of health conditions for each healthy ageing trajectory class sample at baseline for ATHLOS and ELSA, which is one of the studies included in the ATHLOS harmonized cohort. The total number of non-missing cases is used to calculate prevalence. Chronic respiratory diseases may include asthma, CPD, COPD, bronchitis, emphysema.

For both ATHLOS and ELSA, the most prevalent conditions at baseline were hypertension, arterial hypertension, and joint disorders (Fig 3).

### Prevalence of functioning problems

#### ATHLOS

**Fig 4:**
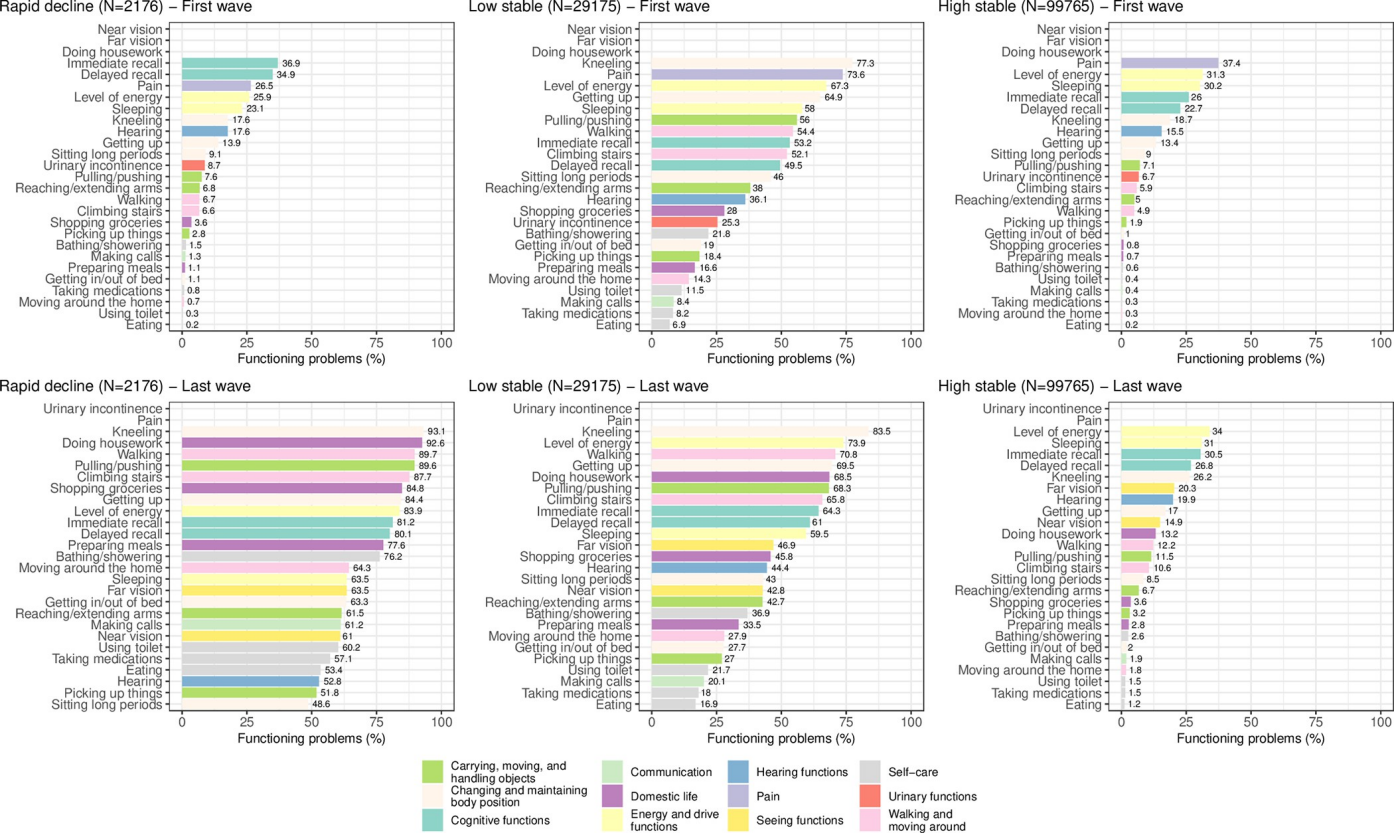
Prevalence of functioning problems at first and last waves for each healthy ageing trajectory class identified for ATHLOS sample.

Fig 4 shows the prevalence of functioning problems at the first and last individual wave of observation. At the last wave of observation, as expected, there is a greater increase of problems of functioning for the ’rapid decline’ trajectory than for the other two trajectories. At the first wave, problems of respondents in the ’high stable’ and ’rapid decline’ trajectories are comparable, the most prevalent problems concerning cognition, pain, as well as energy and drive functions. At the last wave, the prevalence of functioning problems remains similar for the ’high stable’ trajectory. On the opposite, the prevalence of functioning problems of persons in the ’rapid decline’ trajectory increases substantially in all domains, with more than 90% of respondents having problems doing housework and walking.

The bar colors indicate the ICF domains. For each wave, functioning variables with missing bars were not considered for the analysis due to the high number of missing values.

#### ELSA

**Fig 5:**
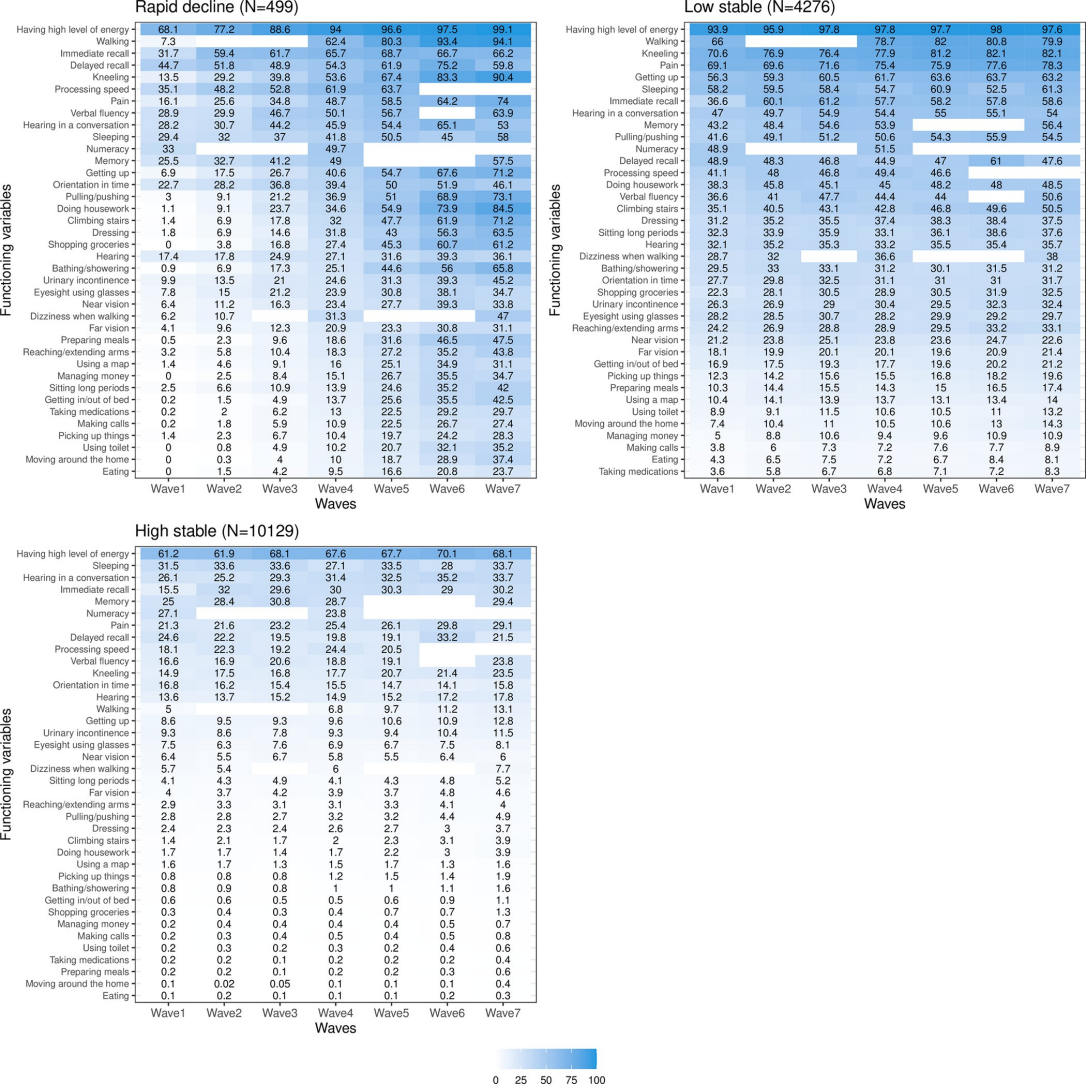
Prevalence of functioning problems at each wave for the healthy ageing trajectory classes identified for ELSA sample, which is one of the studies included in the ATHLOS harmonized cohort. Functioning variables are sorted by the median of the prevalence across the seven waves of the ELSA.

Fig 5 shows that ’level of energy’ and ’sleeping’ are very prevalent problems for all trajectories. Further top domains of prevalent problems are different: changing and maintaining body position and walking and moving around are the very prevalent ones for the ’low stable’ while communication and cognition are the top ones for the ’high stable’ trajectory.

### Expected-influence centrality

#### ATHLOS

**Fig 6:**
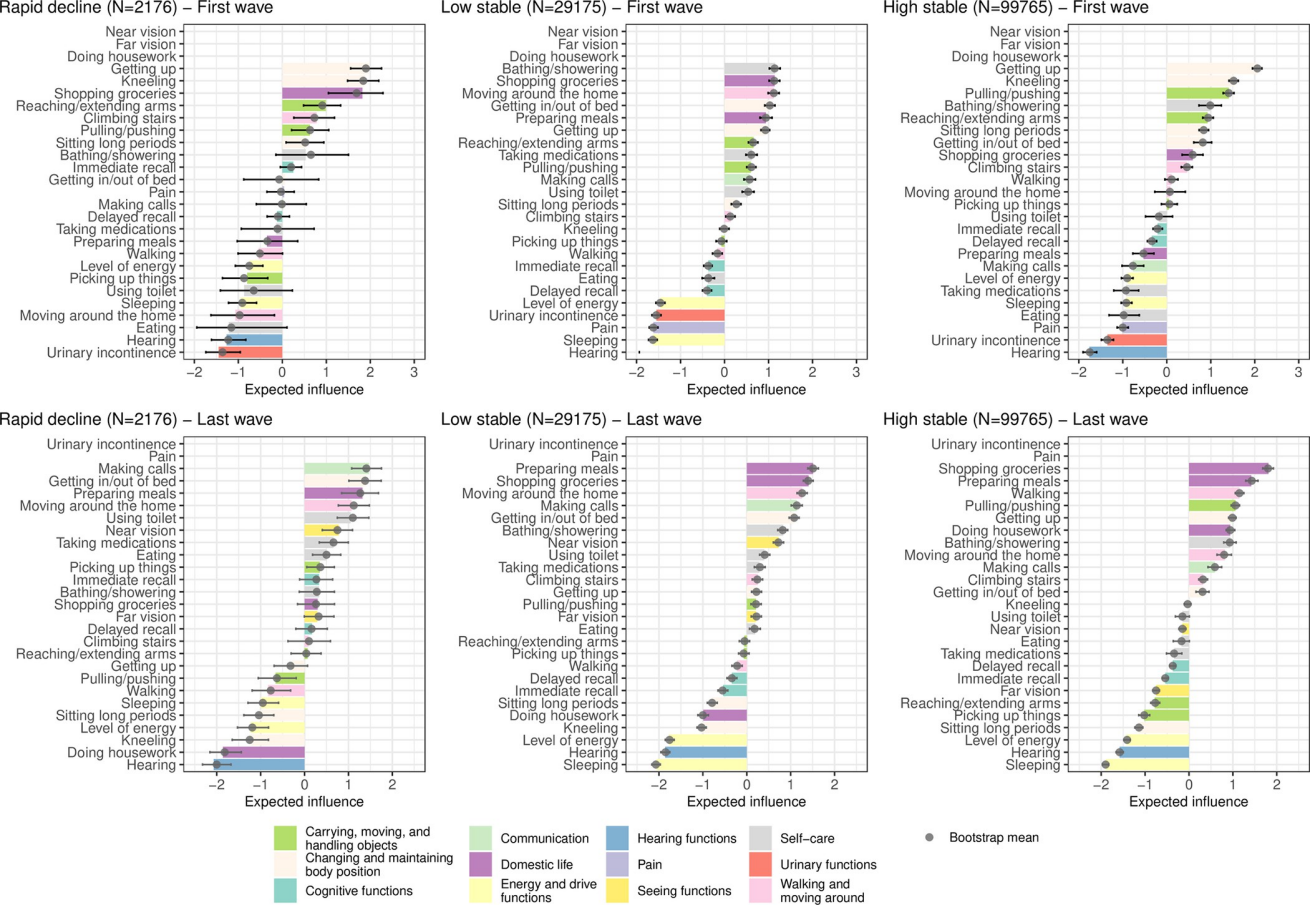
Expected-influence centrality (z-scores) for each functioning variable corresponding to the estimated regularized partial correlation network for each trajectory class identified for the ATHLOS sample. The bars represent the estimates of the original sample, while the grey points represent the bootstrap means with their 2.5% and 97.5% quantiles bars. For each wave, the functioning variables with missing bars were not considered for the analysis due to the high number of missing values.

The expected-influence centrality provides different results than the simple counting of prevalence. S1–S3 Figs show the association structure for each identified healthy ageing trajectory class and the corresponding prevalence of functioning problems. Fig 6 shows that in the first individual wave, two mobility categories and two carrying, moving, and handling objects categories are core functioning categories with the highest connectivity (impact) on overall functioning at the start of the ’rapid decline’ and ’high stable’ trajectory classes. While the domestic life category ’shopping groceries’ has high connectivity in the ’rapid decline’ and the ’low stable’ trajectories, the self-care category ’bathing/showering’ is very relevant for the ’low stable’ and ’high stable’ trajectories. In the last wave, the top three functioning categories with high impact for the ’low stable’ and ’high stable’ trajectory classes are very similar: two domestic life categories, namely ’shopping groceries’ and ’preparing meals’, and one walking and moving around category (’moving around the home’ for ’low stable’ and ’walking’ for the ’high stable’ trajectory). The top functioning categories for the rapid decline’ trajectory include ’preparing meals’, ’getting in/out of bed’ and ’making calls’ (Fig 6).

#### ELSA

**Fig 7:**
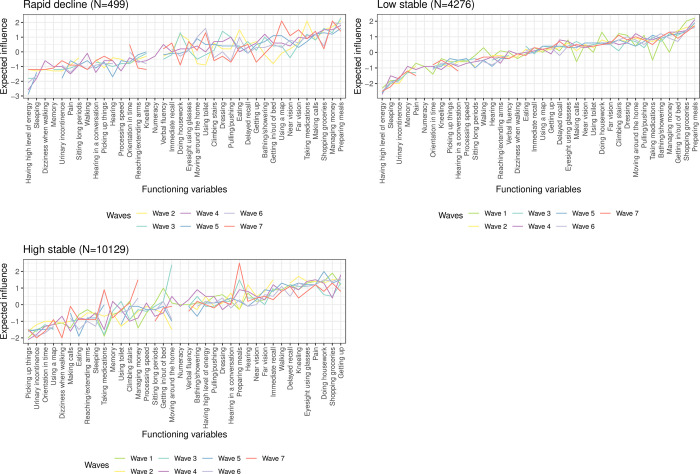
Expected-influence centrality (z-scores) for each functioning variable corresponding to the estimated regularized partial correlation network for each trajectory class across the seven waves of the ELSA sample, which is one of the studies included in the ATHLOS harmonized cohort.

The Fig 7 and S4 Fig show as well that expected-influence centrality and prevalence results are different. Specifically, the domestic life categories ’preparing meals’, ’shopping groceries’ have a high connectivity for the ’rapid decline’ and ’low stable’ trajectory classes, while domestic life functioning categories ’shopping groceries’ and ’doing housework’ have high connectivity for the ’high stable’ trajectory class. While for the ’low stable’ trajectory the expected-influence centrality values follow the same trend over the seven waves, for the other two trajectory classes central functioning variables change from one wave to the next.

Functioning variables are sorted by the median of the expected-influence centrality estimated across the seven waves of the ELSA.

### Bridge expected-influence centrality

#### ATHLOS

**Fig 8:**
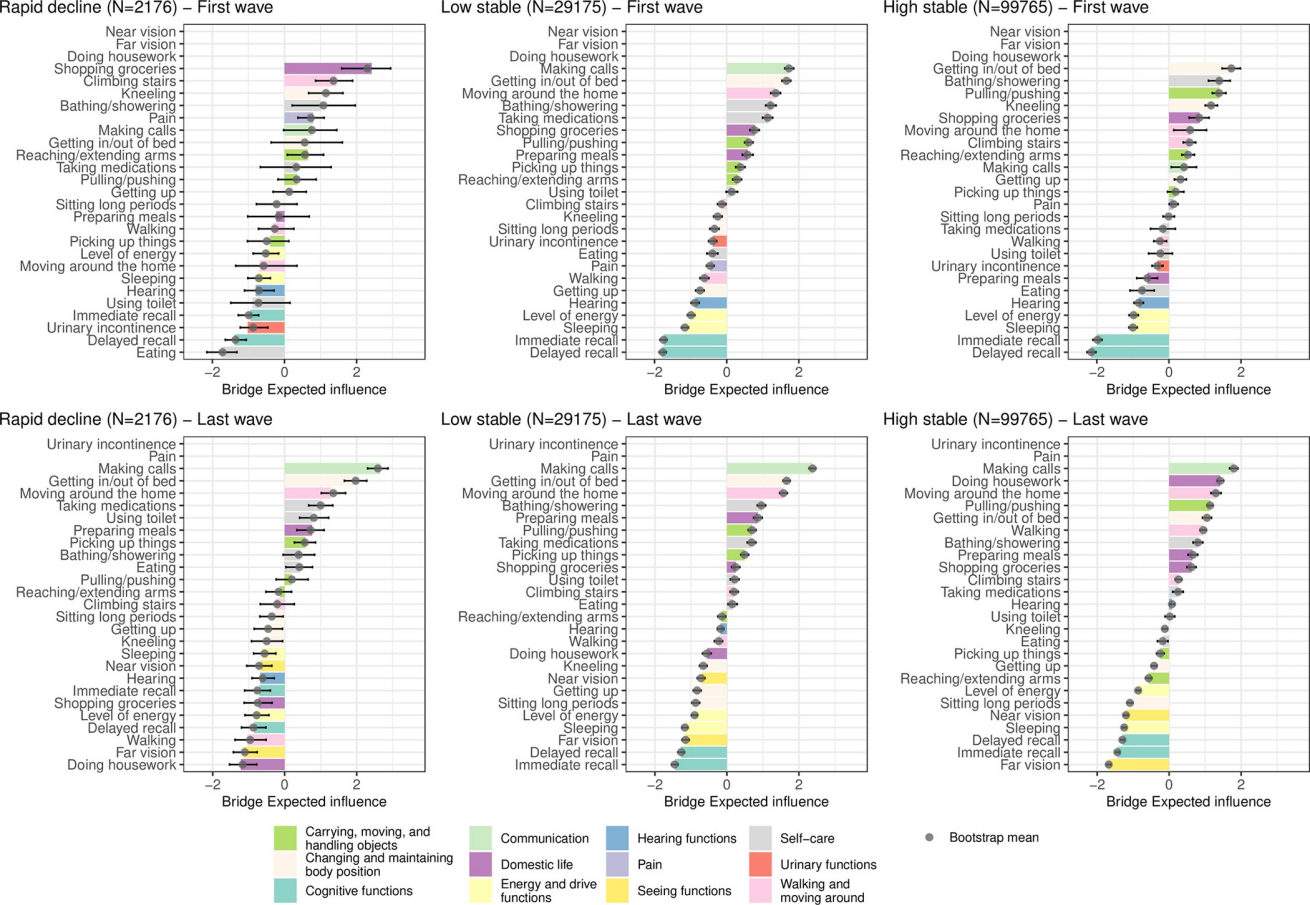
Bridge expected-influence centrality (z-scores) for each functioning variable corresponding to the estimated regularized partial correlation network for each trajectory class identified for the ATHLOS sample. The bars represent the estimates of the original sample, while the grey points represent the bootstrap means with their 2.5% and 97.5% quantiles bars. For each wave, the functioning variable with missing bars were not considered for the analysis due to the high number of missing values.

’Making calls’ is among the most influential functioning categories for all trajectories at the last individual wave and for the ’low stable’ trajectory at the first wave (Fig 8). For the ’rapid decline’ trajectory, at the first wave ’shopping groceries’ is important in connecting the domestic life domain with the remaining functioning domains except urinary functions and pain (Fig 8 and S1 Fig). For the ’high stable’ trajectory at first wave, ’getting in/out of bed’ is important in connecting the changing and maintaining body position domain with the remaining functioning domains except cognitive functions (Fig 8 and S3 Fig).

The stability analysis (S5 Fig) for the first individual wave of the ’rapid decline’ trajectory indicated stability problems. For the last individual wave, good stability was showed. Good stability is shown for both the ’low stable’ and ’high stable’ trajectory classes in general. The same results are shown by the accuracy analysis (S6 Fig).

#### ELSA

**Fig 9:**
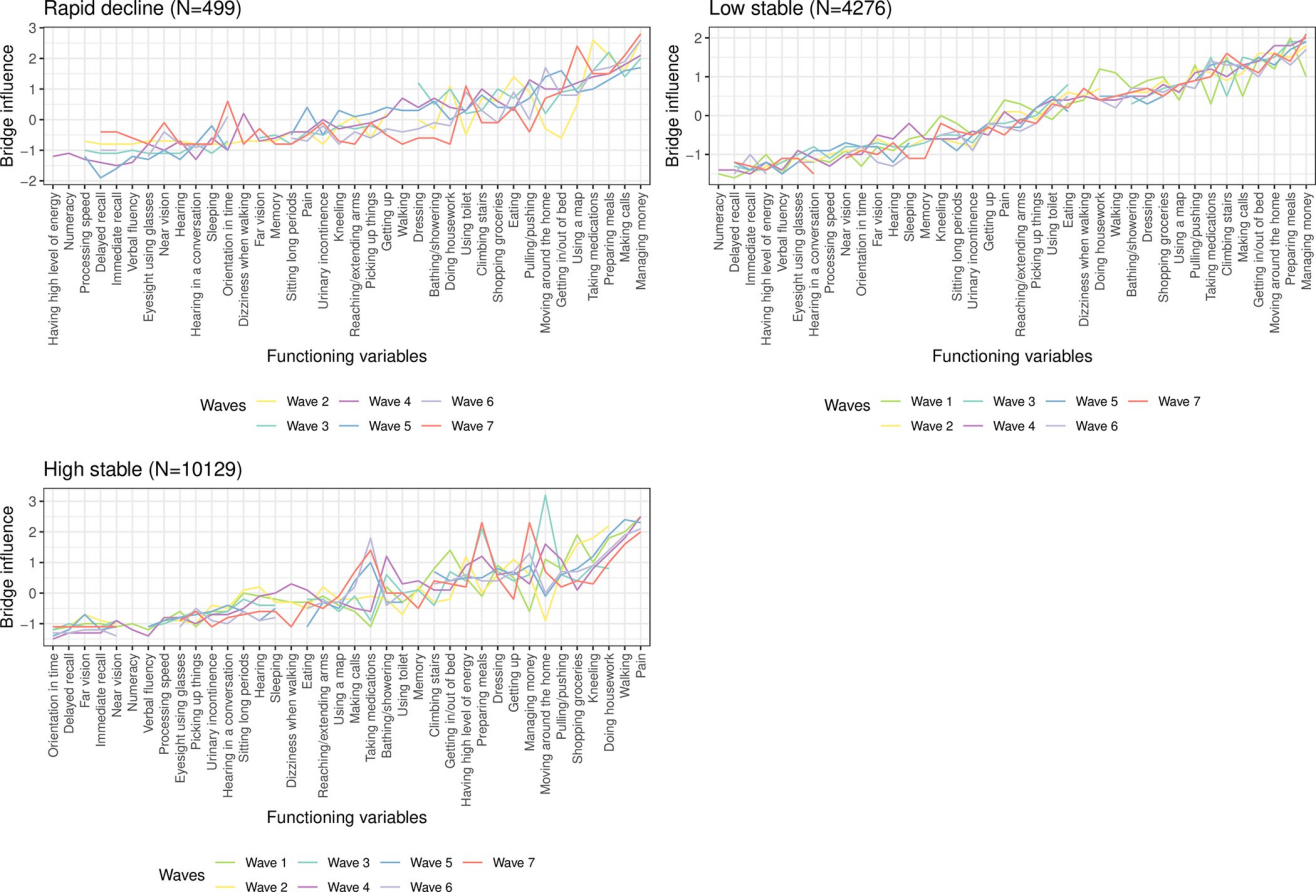
Bridge-influence centrality (z-scores) for each functioning variable corresponding to the estimated regularized partial correlation network for each trajectory class across the seven waves of the ELSA sample, which is one of the studies included in the ATHLOS harmonized cohort. Functioning variables are sorted by the median of the bridge expected-influence centrality estimated across the seven waves of the ELSA.

Fig 9 and S7 Fig show the same trend of the bridge expected-influence centrality for the ’low stable’ trajectory class across the seven waves. ’Making money’ is the most connected functioning variable for the ’rapid decline’ and ’low stable’ trajectory classes, while for the ’high stable’ trajectory, ’pain’ is the most connected one.

The results of the stability of expected-influence estimates are shown in the S8–S10 Figs. While for the ’rapid decline’ trajectory class the correlations between the subsample estimates and the original sample estimates indicate low stability (below 0.5 across all waves), the correlations for the ’high stable’ and ’low stable’ trajectory classes show stability (remained above 0.5). The results of the edge weight accuracy have shown low accuracy (large confidence intervals) for the ’rapid decline’ trajectory class (S11 Fig) and good accuracy (small confidence intervals) for the ’low stable’ and the ’high stable’ trajectory classes (S12 and S13 Figs).

## Discussion

Using the ATHLOS harmonized cohort [9] as well as the methodology previously used to model healthy ageing trajectories with the ATHLOS harmonized cohort [10], we explored whether the trajectories are suitable to identify targets for rehabilitation interventions for the ageing population. At the first individual wave, prevalence of problems of respondents in the ’high stable’ and ’rapid decline’ trajectories are comparable; at the last wave, the prevalence remains similar for the ’high stable’ trajectory but increases substantially in all domains, with more than 90% of respondents having problems doing housework and walking, for persons in the ’rapid decline’ trajectory. Importantly, our study shows that the expected-influence centrality measures provide different results than the prevalence estimates. For instance, in the first wave of data collection mobility and carrying, moving, and handling objects are the functioning domains with the highest impact on overall functioning for the ’rapid decline’ and ’high stable’ trajectories, while the most prevalent functioning problems concern the domains of cognition, pain, as well as energy and drive functions. For evidence- informed planning of rehabilitation services, it is important to identify longitudinal trends considering the context of a country or region. We therefore replicated the analyses using ELSA, which is one of the studies included in the ATHLOS cohort, and observed that the deterioration of the ’rapid decline’ trajectory, for instance, was much less pronounced than in ATHLOS, a rather heterogeneous cohort that includes data from countries with different welfare and health systems. Differences between prevalence and centrality measures of functioning problems in ELSA are comparable to what was observed in ATHLOS. While centrality measures remained consistent for the ’low stable’ trajectory over the seven assessment waves of ELSA, for the ’rapid decline’ and ’high stable’ trajectories centrality changed from one wave to the next, pointing out the importance of using longitudinal data of the context of interest to define what and when rehabilitation services could be considered. Overall, our study provides evidence for the importance of using functioning-oriented approaches, in addition to disease-oriented approaches, to inform the development of rehabilitation programs that meet the needs of a heterogeneous ageing population with distinct ageing trajectories.

Our study is innovative for examining healthy ageing trajectories regarding the prevalence of functioning problems as well as centrality measures. While the prevalence analysis disclosures the functioning categories that contribute most to the decline in functioning over time, the expected-influence centrality measure summarizes how strongly a functioning category is associated with the remaining functioning categories in the regularized partial correlation network. A functioning variable (category) with a high expected-influence may strong or weak association with a specific functioning category. It is important to stress that the observed distribution of response options in the functioning variables may have an impact on the LASSO regression estimates, and thus on the estimation of centrality measures. While the expected-influence centrality focuses on the potential impact of improving functioning in general, the bridge expected-influence centrality focuses on the potential impact of improving functioning in particular functioning domains, such as vision, cognition or mobility, which encompass a group of functioning categories. Potential targets for improving functioning in a selected domain should include functioning categories from the selected domain with the highest prevalence and functioning categories with the highest bridge-expected centrality from the other functioning domains. Combining centrality measures with prevalence is therefore a promising approach for obtaining a full picture of which functioning categories an intervention should potentially target for each of the different healthy ageing trajectories.

Planning rehabilitation provision for the ageing population is a challenge for policy-makers. In part this is because ageing is a complex and heterogeneous process that is not always linked to a single health problem or chronic condition. As the ATHLOS data makes clear, the ageing population is most notably characterized by multimorbidity, rather than by the presence of a single disease or injury [10]. As people age, they accumulate, at often only mild or moderate levels of severity, several functioning problems across body functions, for instance sensory, musculoskeletal or cognitive impairments, and across activities and participation areas, for instance limitations in mobility or self-care and restrictions in their ability to attend social events. Multimorbidity, moreover, is not a matter of dealing with these specific issues separately because impairments, limitations and restrictions interact, synergistically, in complex ways. The interaction results in emergent functioning limitations in areas of people’s life that might not have been predictable from the medical diagnosis alone. We argue, based on our results, that potential rehabilitation services for the ageing population need to have as an entry point functioning and not diseases. However, as shown in two recent scoping reviews, most rehabilitation services delivered to the ageing population are disease-centered [40, 41].

This paper was made possible by the extensive cohort harmonization efforts of the EU project ATHLOS. Moving forward, creating and using heathy ageing trajectories to plan rehabilitation strategies for the ageing population will require routinely collecting, ideally through health information systems (HIS), and routinely analyzing functioning data using the ageing trajectories approach [42]. The need for functioning data is one of the reasons why a key action of the WHO Rehabilitation 2030 initiative is to reshape and expand the scope of HIS at the national level to accommodate the routine collection of functioning data at clinical, services and population level. Data on functioning collected in clinical practice is especially important for rehabilitation planning and delivery at the micro and meso level: it supports targeted goal-setting between users and health care professionals, facilitates the monitoring of expected outcomes, and, when aggregated at facility or higher levels, can provide guidance for evidence-informed service allocation and financing [43]. Importantly, data collected in clinical practice can support the prediction of the ageing trajectory of a person and guide the selection of targeted interventions to ensure, as requested by WHO, the optimization of functioning that enables well-being in older age.

The role of rehabilitation in achieving the goals of the healthy ageing agenda has yet to be fully appreciated by policy-makers. Current estimates show that around 2.4 billion persons live with health problems that could benefit from rehabilitation at some point in life, most of them belonging to the ageing population [6]. As WHO has made clear, the ageing process is essentially a matter of declines in intrinsic capacity that are played out in people’s lives as declines in the performance of their daily life, that is, in functioning [3]. The aim of rehabilitation is to optimize functioning in people’s lives and rehabilitation interventions target either the improvement, maintaining or slowing down of declines in functioning for people with health conditions, in light of their environmental context [4]. This may involve restorative or compensatory therapeutic strategies, education for self-management, the provision and follow-up of assistive technologies, or environmental adaptations in the home, among others. Especially in older ages, rehabilitation can improve functioning limitations linked to a range of age-related declines in intrinsic capacity, including those associated with NCDs. In short, it is clear that rehabilitation interventions can be an important health strategy in achieving the goals of the UN Decade of Ageing [44]. Yet while failing to integrate rehabilitation into the healthy ageing agenda would be a lost opportunity [7], an open question is how rehabilitation should be planned at population level in light of heterogeneous ageing trajectories presented by WHO in the WRA [3] and demonstrated in ATLHOS using a very large harmonized cohort [9].

There are several limitations in our paper. Firstly, as the ATHLOS and ELSA ‘rapid decline’ samples indicate, there is only moderate stability and low stability respectively for expected-influence estimates, and these estimates should therefore be interpreted with caution. Secondly, for the ATHLOS sample, because of missing cases for many functioning variables, we could not consider all 41 variables that correspond to the respective items that were used to construct the HAI. For example, while pain showed higher prevalence for the first individual wave, it was not included in the last wave due to missing data. Thirdly, the selection of time points of interest in ATHLOS (individual first and last wave) was based on the available data and not based on their meaningfulness in relation to rehabilitation intervention targets. Future approaches should be looking at specific and meaningful time points of interest (e.g., onset of a chronic health condition or other events such as falls, moving to a nursing care facility). Changes in the centrality of functioning variables over time for the ELSA sample indicate that rehabilitation interventions should address different aspects of functioning over time. Fourthly, in the RPCN we could observed negative edge weights for associations that we would have expected to be positive. This might point to the presence of common effect relationships in the network estimation. Fifthly, the use of the ELSA sample, which represents only 10% of the ATHLOS sample, to identify intervention targets at country level may lead to biased results. However, the ELSA sample is part of the ATHLOS sample and was used in the development of the HAI index. Sixthly, the sample size of the ATHLOS harmonized cohort sample decreases at the last individual wave due to the high dropout rate where information on functioning is collected over 11 waves (the ALSA and HRS cohort studies). Consequently, survival bias may be present in the selected samples for our analyses. Despite these limitations, our findings offer insights on how healthy ageing trajectories can be used to inform rehabilitation planning at the population level.

### Conclusion

By exploring whether healthy ageing trajectories are suitable to identify targets for rehabilitation interventions for the ageing population, our study stressed the relevance, especially for the ’rapid decline’ trajectory, of providing rehabilitation interventions that match the most prevalent and central functioning problems of s specific trajectory. We also showed that centrality measures provide different results than prevalence estimates, and that healthy ageing trajectories may be a much more nuanced and relevant way to define what are the rehabilitation targets needed by a heterogeneous ageing population. The routine modelling and use of healthy ageing trajectories at country level has the potential to make a meaningful contribution to the planning and delivery of rehabilitation interventions through health systems and to informed policy making.

## Supporting information

S1 Table List of ICF domains and functioning variables based on the 41 items used in the development of the healthy ageing index. (DOCX)

S1 Fig Regularized partial correlation network returned by the graphical LASSO, showing associations between pairs of functioning variables for the ’rapid decline’ trajectory class for the first and last wave of the ATHLOS sample. The dash line indicates negative partial correlation. The thicker the edge is, the higher the estimated partial correlation. The colour of the indicates the ICF domains. The size of the node indicates the prevalence of the functioning problem, using median as a cutting point.
(PDF)

S2 Fig Regularized partial correlation network returned by the graphical LASSO, showing associations between pairs of functioning variables for the ‘low stable’ trajectory class for the first and last wave of the ATHLOS sample. The dash line indicates negative partial correlation. The thicker the edge is, the higher the estimated partial correlation. The colour of the nodes indicates the ICF domains. The size of the node indicates the prevalence of the functioning problem, using median as a cutting point.
(PDF)

S3 Fig Regularized partial correlation network returned by the graphical LASSO, showing associations between pairs of functioning variables for the ‘high stable’ trajectory class for the first and last wave of the ATHLOS sample. The dash line indicates negative partial correlation. The thicker the edge is, the higher the estimated partial correlation. The colour of the indicates the ICF domains. The size of the node indicates the prevalence of the functioning problem, using median as a cutting point.
(PDF)

S4 Fig Heat map showing the values of the expected-influence centrality (z-scores) for each functioning variable corresponding to the estimated regularized partial correlation network for each trajectory class across the seven waves of the ELSA sample, which is one of the studies included in the ATHLOS harmonized cohort. (PDF)

S5 Fig Stability of expected-influence estimates for each trajectory class at the first and the last wave of the ATHLOS sample. (PDF)

S6 Fig Edge accuracy graph in the non-parametric accuracy analysis for the regularized partial correlation network returned via the graphical LASSO depicting associations between pairs of functioning variables for each trajectory class for the first and last wave of the ELSA sample, which is one of the studies included in the ATHLOS harmonized cohort. The x-axis represents the edges’ weights, while every line on the y-axis represents a specific edge (not shown). The red line shows the estimate of the edge weights for each wave, and the grey bars the 95% confidence intervals of the bootstrap means of edge weights.
(PDF)

S7 Fig Heat map showing the values of the bridge expected-influence centrality (z-scores) for each functioning variable corresponding to the estimated regularized partial correlation network for each trajectory class across the seven waves of the ELSA sample, which is one of the studies included in the ATHLOS harmonized cohort. (PDF)

S8 Fig Stability of expected-influence centrality estimates for ’rapid decline’ trajectory class at all seven waves of the ELSA sample, which is one of the studies included in the ATHLOS harmonized cohort. (PDF)

S9 Fig Stability of expected-influence centrality estimates for ’low stable’ trajectory class at all seven waves of the ELSA sample, which is one of the studies included in the ATHLOS harmonized cohort. (PDF)

S10 Fig Stability of expected-influence centrality estimates for ’high stable’ trajectory class at all seven waves of the ELSA sample, which is one of the studies included in the ATHLOS harmonized cohort. (PDF)

S11 Fig Edge accuracy graph in the non-parametric accuracy analysis for the regularized partial correlation network returned via the graphical LASSO depicting associations between pairs of functioning variables for ’rapid decline’ trajectory class for all seven waves of the ELSA sample, which is one of the studies included in the ATHLOS harmonized cohort. The x-axis represents the edges’ weights, while every line on the y-axis represents a specific edge (not shown). The red line shows the estimate of the edge weights for each wave, and the grey bars the 95% confidence intervals of the bootstrap means of edge weights.
(PDF)

S12 Fig Edge accuracy graph in the non-parametric accuracy analysis for the regularized partial correlation network returned via the graphical LASSO depicting associations between pairs of functioning variables for ’low stable’ trajectory class for all seven waves of the ELSA sample, which is one of the studies included in the ATHLOS harmonized cohort. The x-axis represents the edges’ weights, while every line on the y-axis represents a specific edge (not shown). The red line shows the estimate of the edge weights for each wave, and the grey bars the 95% confidence intervals of the bootstrap means of edge weights.
(PDF)

S13 Fig Edge accuracy graph in the non-parametric accuracy analysis for the regularized partial correlation network returned via the graphical LASSO depicting associations between pairs of functioning variables for ’high stable’ trajectory class for all seven waves of the ELSA sample, which is one of the studies included in the ATHLOS harmonized cohort. The x-axis represents the edges’ weights, while every line on the y-axis represents a specific edge (not shown). The red line shows the estimate of the edge weights for each wave, and the grey bars the 95% confidence intervals of the bootstrap means of edge weights.
(PDF)
