# Supplementary material for: Are healthy ageing trajectories suitable to identify rehabilitation needs of the ageing population? An exploratory study using ATHLOS cohort data
Source: PLoS One. 2024 Jul 9;19(7):e0303865. doi: 10.1371/journal.pone.0303865 (PMC11232974; doi:10.1371/journal.pone.0303865)
Supplement: S1 Table — (DOCX) [file pone.0303865.s001.docx]

| **ICF domains** | **Functioning variables** | **Study labels** |
| --- | --- | --- |
| Cognitive functions | Memory | Memory |
| Cognitive functions | Immediate recall | Immediate recall |
| Cognitive functions | Delayed recall | Delayed recall |
| Cognitive functions | Verbal fluency | Verbal fluency |
| Cognitive functions | Orientation in time | Orientation time |
| Cognitive functions | Processing speed | Processing speed |
| Cognitive functions | Using a map | Using a map |
| Cognitive functions | Numeracy | Numeracy |
| Energy and drive functions | Sleeping | Sleeping |
| Energy and drive functions | Having high level of energy | Having high level of energy |
| Urinary functions | Urinary incontinence | Urinary incontinence |
| Pain | Experiences some degree of pain | Pain |
| Seeing functions | Near vision | Near vision |
| Seeing functions | Far vision | Far vision |
| Seeing functions | Eyesight using glasses or lens as usual | Eyesight with glasses |
| Hearing functions | Hearing in general | Hearing |
| Hearing functions | Hearing in a conversation | Hearing in a conversation |
| Communication | Making telephone calls | Making calls |
| Changing and maintaining body position | Stooping, kneeling or crouching | Kneeling |
| Changing and maintaining body position | Getting up from sitting down | Getting up |
| Changing and maintaining body position | Sitting for long periods | Sitting long periods |
| Changing and maintaining body position | Getting in or out of bed | Getting in/out of bed |
| Carrying, moving and handling objects | Lifting or carrying weights | Lifting or carrying weights |
| Carrying, moving and handling objects | Pulling or pushing large objects | Pulling/pushing |
| Carrying, moving and handling objects | Reaching or extending arms | Reaching or extending arms |
| Carrying, moving and handling objects | Picking up things with fingers | Picking up things |
| Walking and moving around | Walking by yourself and without any equipment | Walking |
| Walking and moving around | Walking speed | Walking speed |
| Walking and moving around | Dizziness when walking on a level surface | Dizziness when walking |
| Walking and moving around | Climbing stairs | Climbing stairs |
| Walking and moving around | Moving around the home | Moving around home |
| Walking and moving around | Getting out of the house | Getting out house |
| Self-care | Bathing or showering | Bathing or showering |
| Self-care | Getting dressed | Getting dressed |
| Self-care | Taking medications | Taking medications |
| Self-care | Using the toilet | Using toilet |
| Self-care | Eating | Eating |
| Domestic life | Doing housework | Doing housework |
| Domestic life | Shopping for groceries | Shopping groceries |
| Domestic life | Difficulties in preparing meals | Preparing meals |
| Economic life | Managing money, bills or expenses | Managing money |

**S1 Table. List of ICF domains and functioning variables based on the 41 items used in the development of the healthy ageing index.**
