# Supplementary material for: Are healthy ageing trajectories suitable to identify rehabilitation needs of the ageing population? An exploratory study using ATHLOS cohort data
Source: PLoS One. 2024 Jul 9;19(7):e0303865. doi: 10.1371/journal.pone.0303865 (PMC11232974; doi:10.1371/journal.pone.0303865)
Supplement: S1 Fig — The dash line indicates negative partial correlation. The thicker the edge is, the higher the estimated partial correlation. The colour of the indicates the ICF domains. The size of the node indicates the prevalence of the functioning problem, using median as a cutting point. (PDF) [file pone.0303865.s002.pdf]

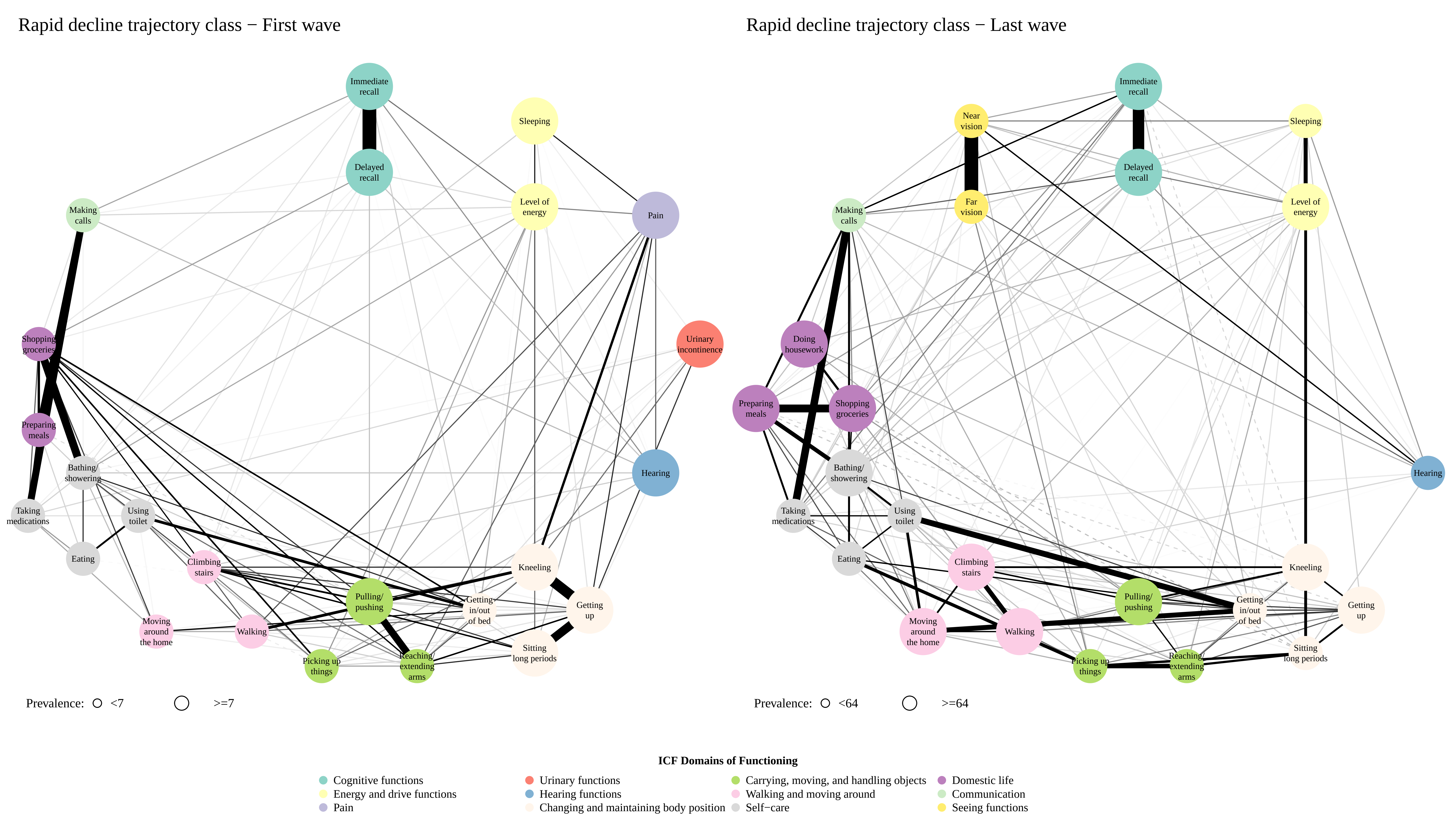
Rapid decline trajectory class − First wave
Immediate recall
Delayed recall
Sleeping
Level of energy
Pain
Making calls
Urinary incontinence
Shopping groceries
Preparing meals
Bathing/ showering
Hearing
Taking medications
Using toilet
Eating
Climbing stairs
Kneeling
Pulling/ pushing
Getting in/out of bed
Getting up
Moving around the home
Walking
Sitting long periods
Picking up things
Reaching/ extending arms
Prevalence: <7 >=7
Rapid decline trajectory class − Last wave
Immediate recall
Near vision
Sleeping
Delayed recall
Far vision
Level of energy
Making calls
Doing housework
Preparing meals
Shopping groceries
Bathing/ showering
Hearing
Taking medications
Using toilet
Eating
Climbing stairs
Kneeling
Pulling/ pushing
Getting in/out of bed
Getting up
Moving around the home
Walking
Sitting long periods
Picking up things
Reaching/ extending arms
Prevalence: <64 >=64
ICF Domains of Functioning
Cognitive functions
Energy and drive functions
Pain
Urinary functions
Hearing functions
Changing and maintaining body position
Carrying, moving, and handling objects
Walking and moving around
Self−care
Domestic life
Communication
Seeing functions
