# Supplementary material for: Are healthy ageing trajectories suitable to identify rehabilitation needs of the ageing population? An exploratory study using ATHLOS cohort data
Source: PLoS One. 2024 Jul 9;19(7):e0303865. doi: 10.1371/journal.pone.0303865 (PMC11232974; doi:10.1371/journal.pone.0303865)
Supplement: S4 Fig — (PDF) [file pone.0303865.s005.pdf]

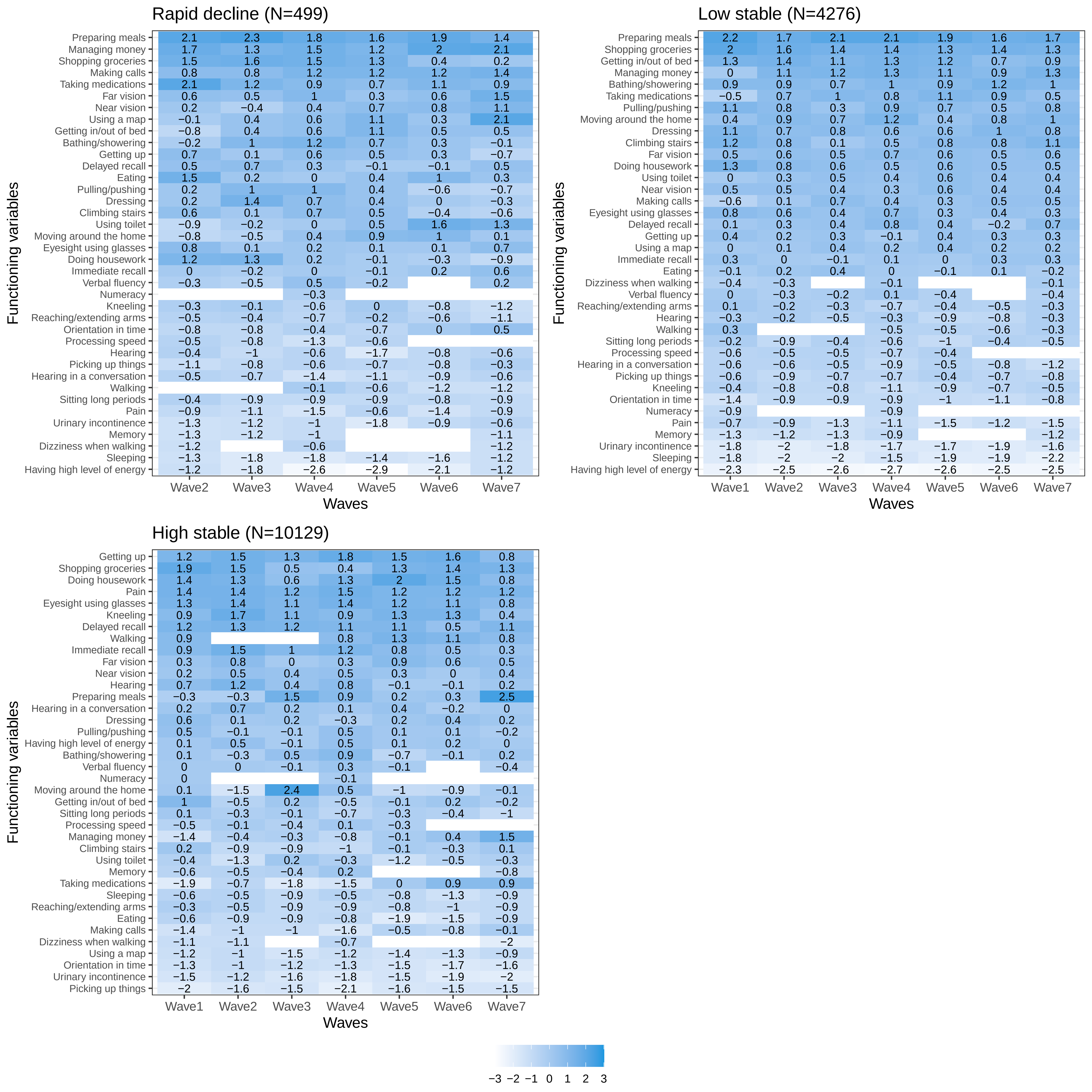

Rapid decline (N=499)
Preparing meals
Managing money
Shopping groceries
Making calls
Taking medications
Far vision
Near vision
Using a map
Getting in/out of bed
Bathing/showering
Getting up
Delayed recall
Eating
Pulling/pushing
Dressing
Climbing stairs
Using toilet
Moving around the home
Eyesight using glasses
Doing housework
Immediate recall
Verbal fluency
Numeracy
Kneeling
Reaching/extending arms
Orientation in time
Processing speed
Hearing
Picking up things
Hearing in a conversation
Walking
Sitting long periods
Pain
Urinary incontinence
Memory
Dizziness when walking
Sleeping
Having high level of energy
Wave2 Wave3 Wave4 Wave5 Wave6 Wave7
Waves
Functioning variables
Low stable (N=4276)
Preparing meals
Shopping groceries
Getting in/out of bed
Managing money
Bathing/showering
Taking medications
Pulling/pushing
Moving around the home
Dressing
Climbing stairs
Far vision
Doing housework
Using toilet
Near vision
Making calls
Eyesight using glasses
Delayed recall
Getting up
Using a map
Immediate recall
Eating
Dizziness when walking
Verbal fluency
Reaching/extending arms
Hearing
Walking
Sitting long periods
Processing speed
Hearing in a conversation
Picking up things
Kneeling
Orientation in time
Numeracy
Pain
Memory
Urinary incontinence
Sleeping
Having high level of energy
Wave1 Wave2 Wave3 Wave4 Wave5 Wave6 Wave7
Waves
Functioning variables
High stable (N=10129)
Getting up
Shopping groceries
Doing housework
Pain
Eyesight using glasses
Kneeling
Delayed recall
Walking
Immediate recall
Far vision
Near vision
Hearing
Preparing meals
Hearing in a conversation
Dressing
Pulling/pushing
Having high level of energy
Bathing/showering
Verbal fluency
Numeracy
Moving around the home
Getting in/out of bed
Sitting long periods
Processing speed
Managing money
Climbing stairs
Using toilet
Memory
Taking medications
Sleeping
Reaching/extending arms
Eating
Making calls
Dizziness when walking
Using a map
Orientation in time
Urinary incontinence
Picking up things
Wave1 Wave2 Wave3 Wave4 Wave5 Wave6 Wave7
Waves
Functioning variables
−3 −2 −1 0 1 2 3
