# Supplementary material for: Are healthy ageing trajectories suitable to identify rehabilitation needs of the ageing population? An exploratory study using ATHLOS cohort data
Source: PLoS One. 2024 Jul 9;19(7):e0303865. doi: 10.1371/journal.pone.0303865 (PMC11232974; doi:10.1371/journal.pone.0303865)
Supplement: S5 Fig — (PDF) [file pone.0303865.s006.pdf]

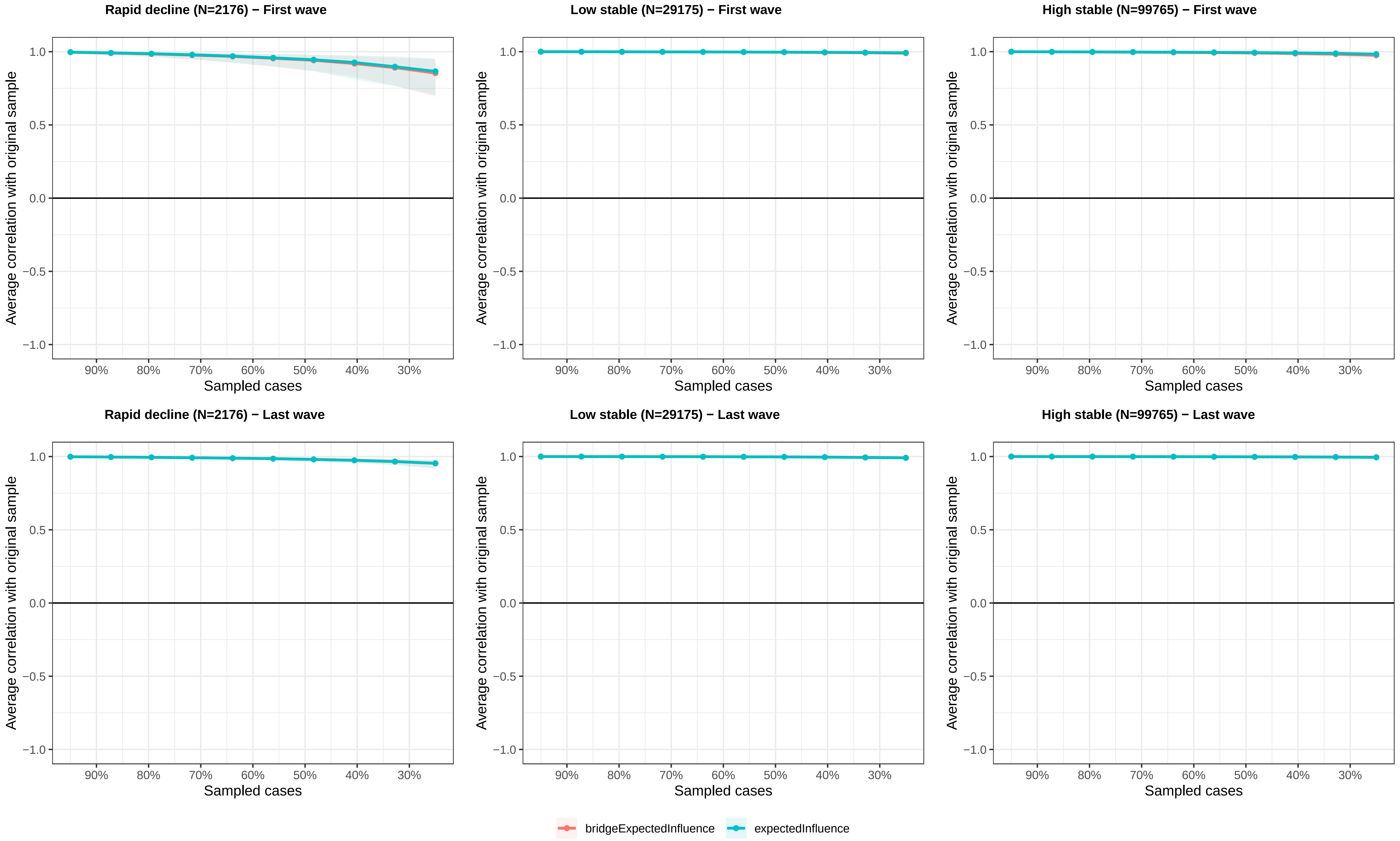
Rapid decline (N=2176) − First wave
Low stable (N=29175) − First wave
High stable (N=99765) − First wave
Rapid decline (N=2176) − Last wave
Low stable (N=29175) − Last wave
High stable (N=99765) − Last wave
Average correlation with original sample
Sampled cases
1.0
0.5
0.0
−0.5
−1.0
90%
80%
70%
60%
50%
40%
30%
bridgeExpectedInfluence
expectedInfluence
