# Supplementary material for: Are healthy ageing trajectories suitable to identify rehabilitation needs of the ageing population? An exploratory study using ATHLOS cohort data
Source: PLoS One. 2024 Jul 9;19(7):e0303865. doi: 10.1371/journal.pone.0303865 (PMC11232974; doi:10.1371/journal.pone.0303865)
Supplement: S6 Fig — The x-axis represents the edges’ weights, while every line on the y-axis represents a specific edge (not shown). The red line shows the estimate of the edge weights for each wave, and the grey bars the 95% confidence intervals of the bootstrap means of edge weights. (PDF) [file pone.0303865.s007.pdf]

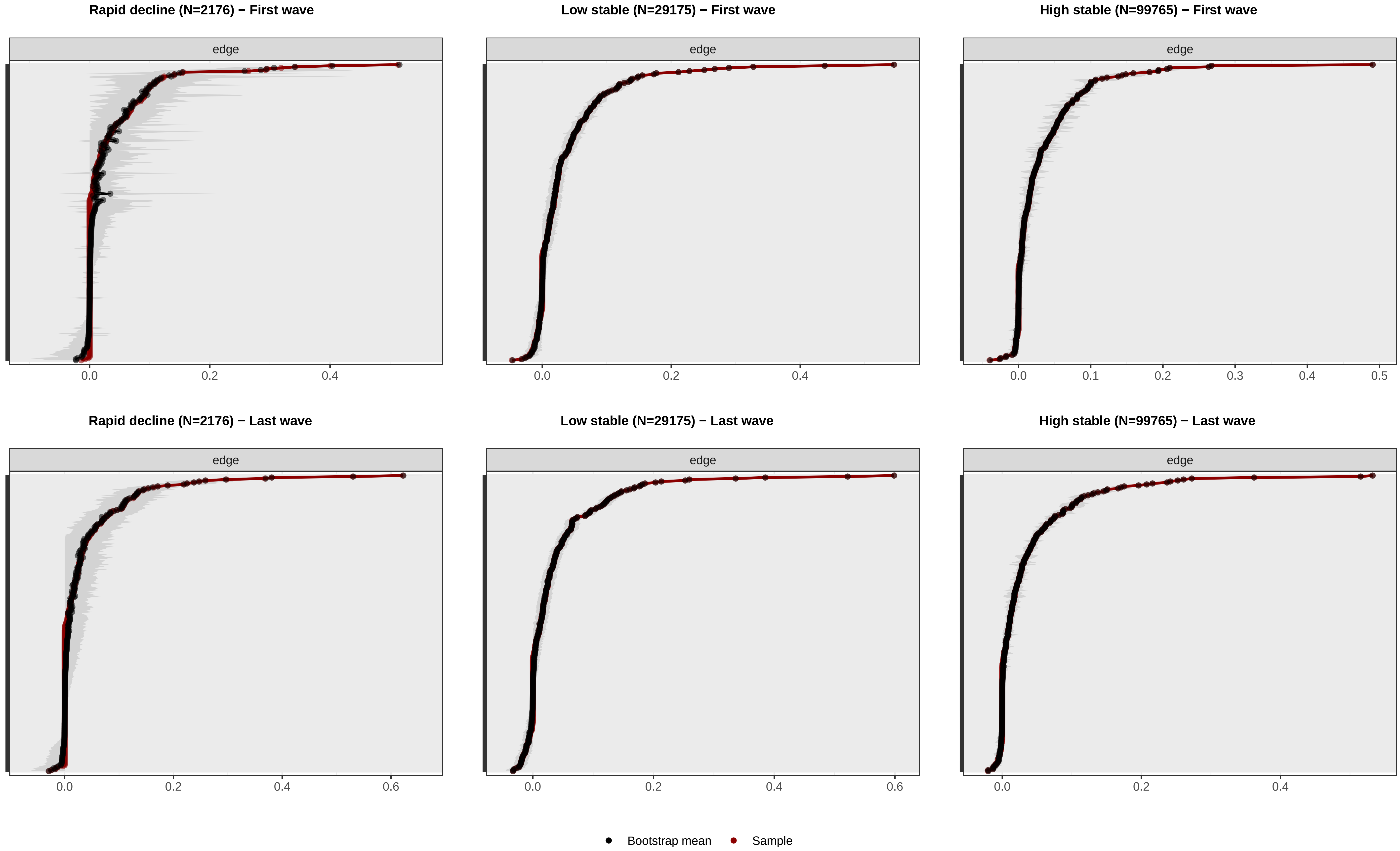

Rapid decline (N=2176) − First wave
Low stable (N=29175) − First wave
High stable (N=99765) − First wave
edge
0.0
0.2
0.4
0.1
0.3
0.5
Rapid decline (N=2176) − Last wave
Low stable (N=29175) − Last wave
High stable (N=99765) − Last wave
0.6
Bootstrap mean
Sample
