# Supplementary figures and images for: Are healthy ageing trajectories suitable to identify rehabilitation needs of the ageing population? An exploratory study using ATHLOS cohort data
Source: PLoS One. 2024 Jul 9;19(7):e0303865. doi: 10.1371/journal.pone.0303865 (PMC11232974; doi:10.1371/journal.pone.0303865)

Rapid decline (N=499)

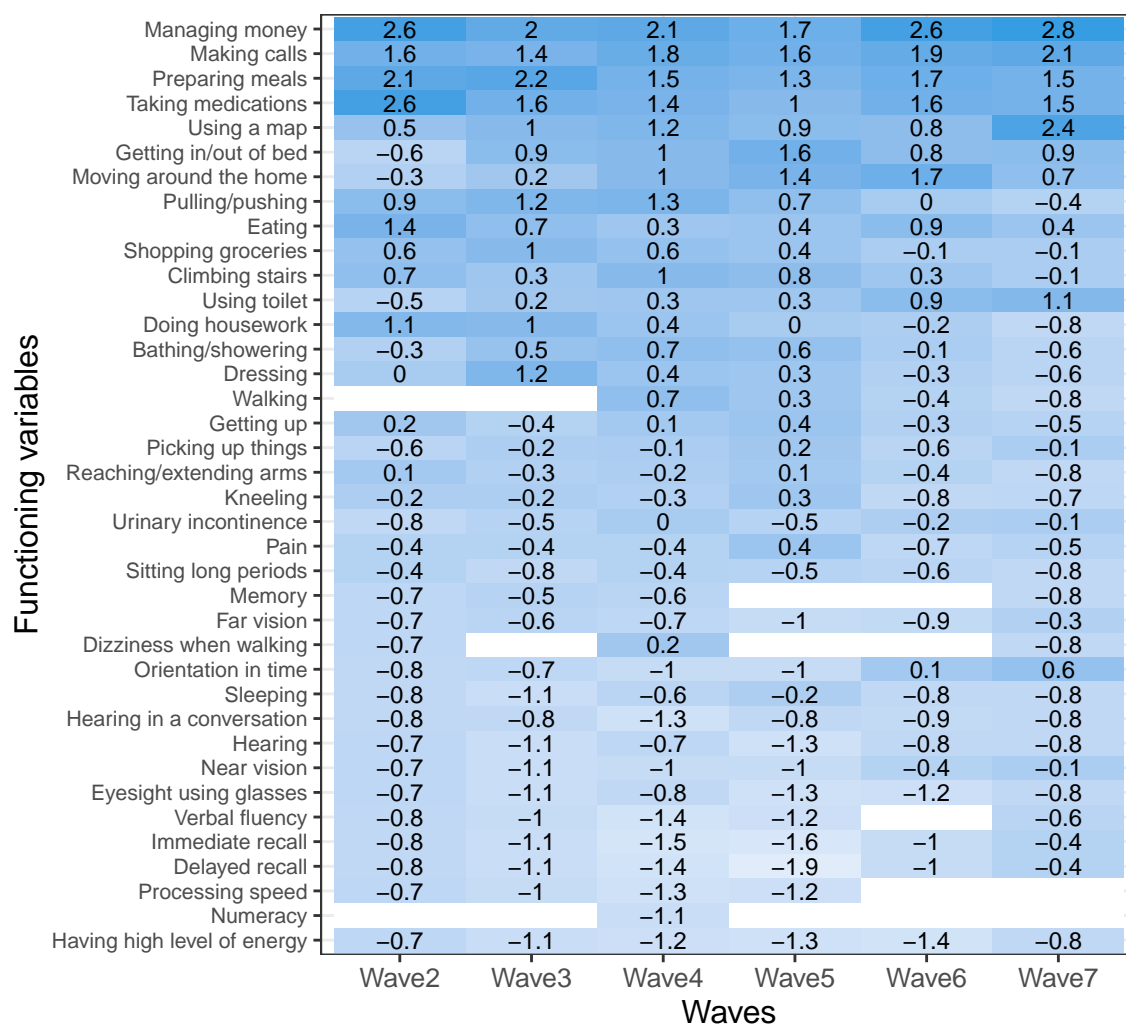

Low stable (N=4276)

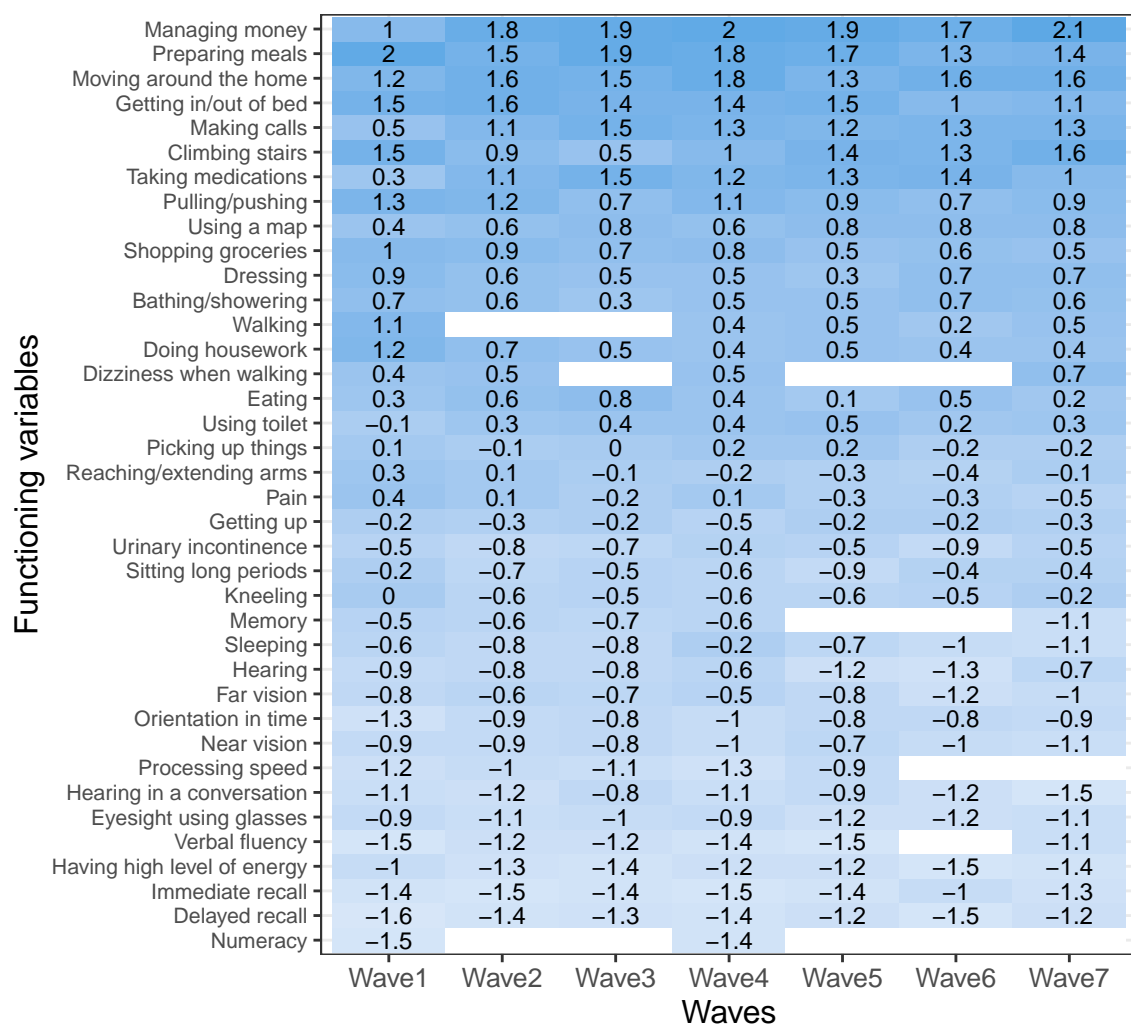

High stable (N=10129)

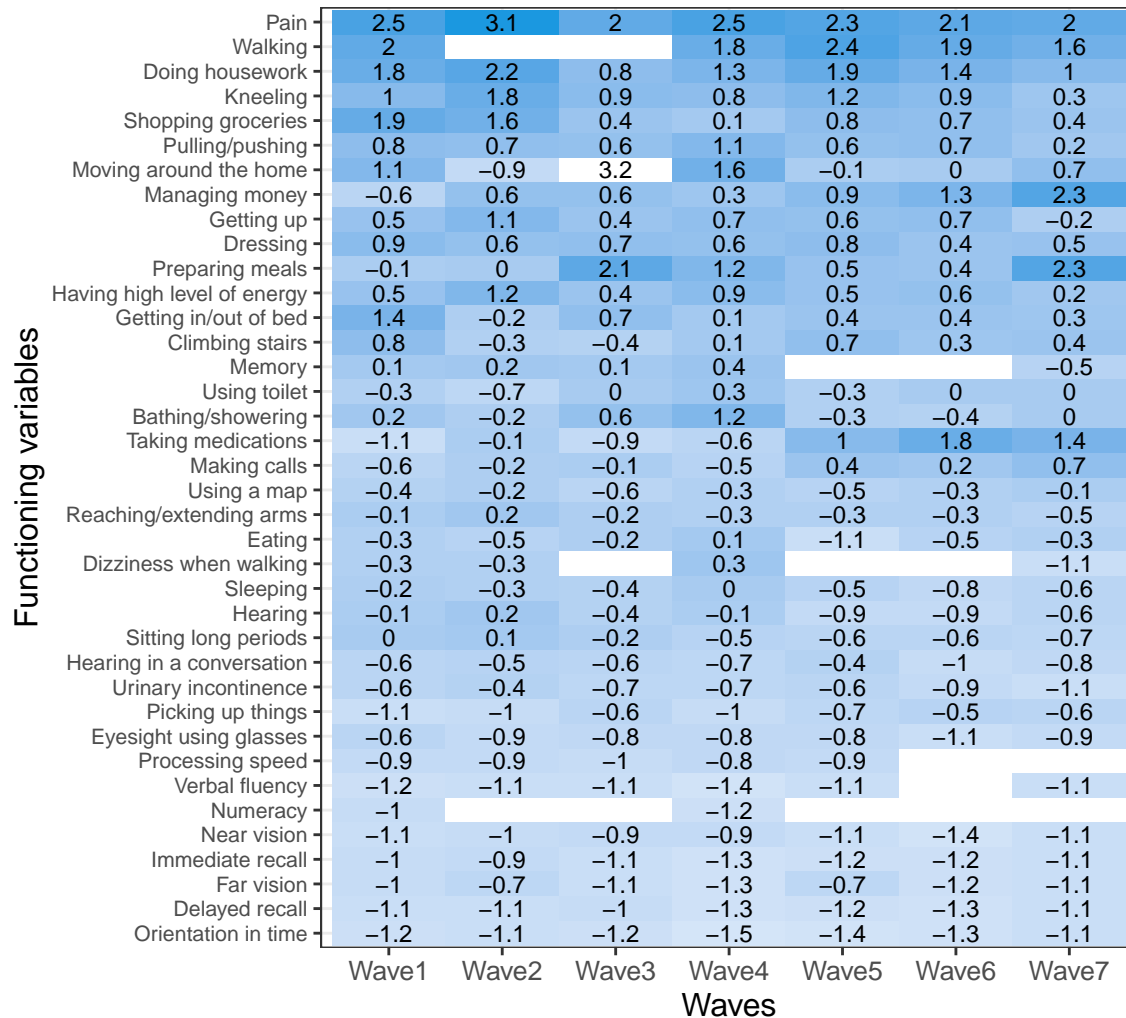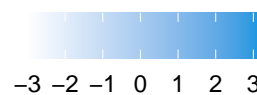

Supplement: S7 Fig — (PDF) [file pone.0303865.s008.pdf]
