# Supplementary material for: Are healthy ageing trajectories suitable to identify rehabilitation needs of the ageing population? An exploratory study using ATHLOS cohort data
Source: PLoS One. 2024 Jul 9;19(7):e0303865. doi: 10.1371/journal.pone.0303865 (PMC11232974; doi:10.1371/journal.pone.0303865)
Supplement: S8 Fig — (PDF) [file pone.0303865.s009.pdf]

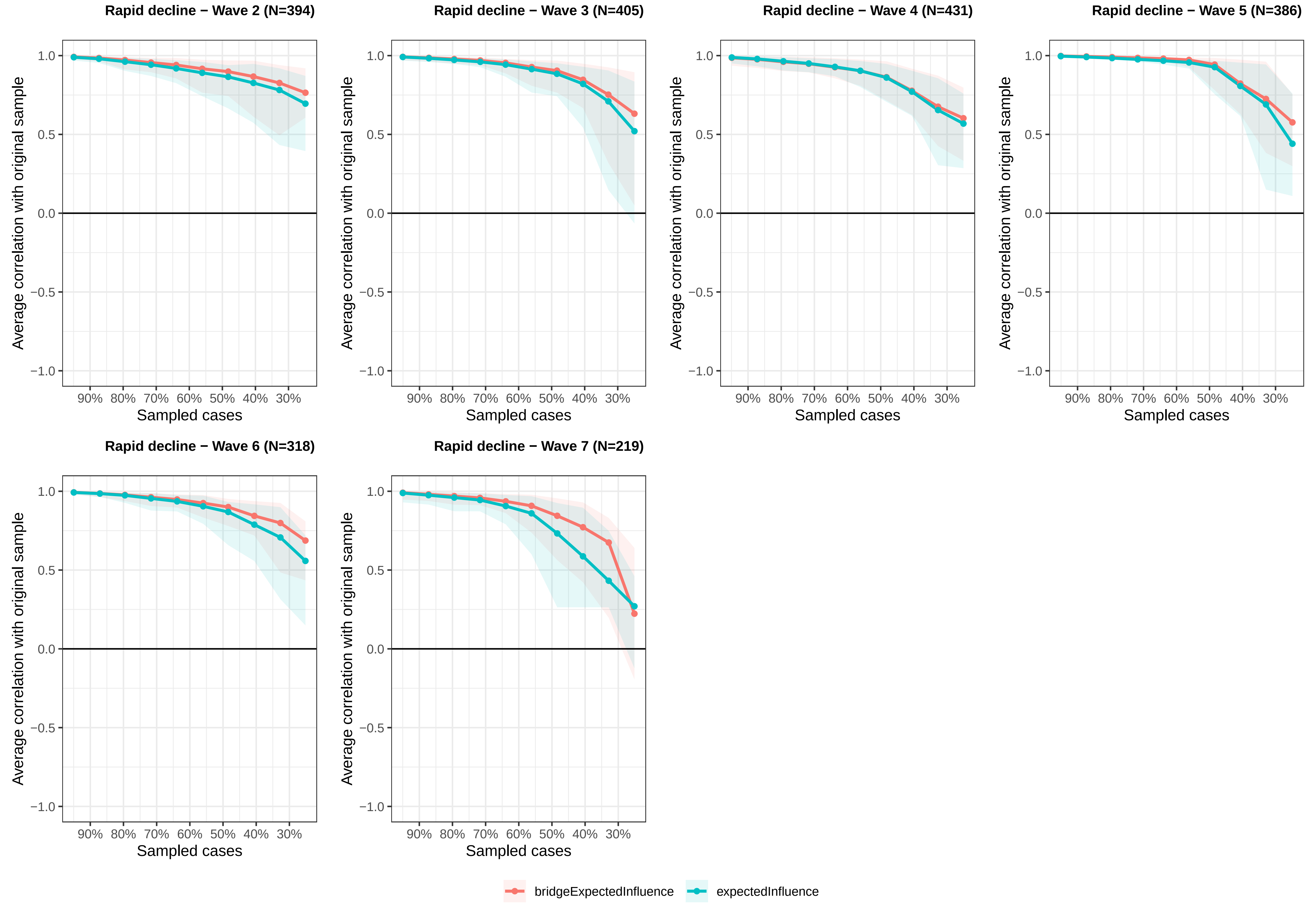

Rapid decline − Wave 2 (N=394)
Rapid decline − Wave 3 (N=405)
Rapid decline − Wave 4 (N=431)
Rapid decline − Wave 5 (N=386)
Rapid decline − Wave 6 (N=318)
Rapid decline − Wave 7 (N=219)
Average correlation with original sample
1.0
0.5
0.0
−0.5
−1.0
90%
80%
70%
60%
50%
40%
30%
Sampled cases
bridgeExpectedInfluence
expectedInfluence
