# Supplementary material for: Are healthy ageing trajectories suitable to identify rehabilitation needs of the ageing population? An exploratory study using ATHLOS cohort data
Source: PLoS One. 2024 Jul 9;19(7):e0303865. doi: 10.1371/journal.pone.0303865 (PMC11232974; doi:10.1371/journal.pone.0303865)
Supplement: S10 Fig — (PDF) [file pone.0303865.s011.pdf]

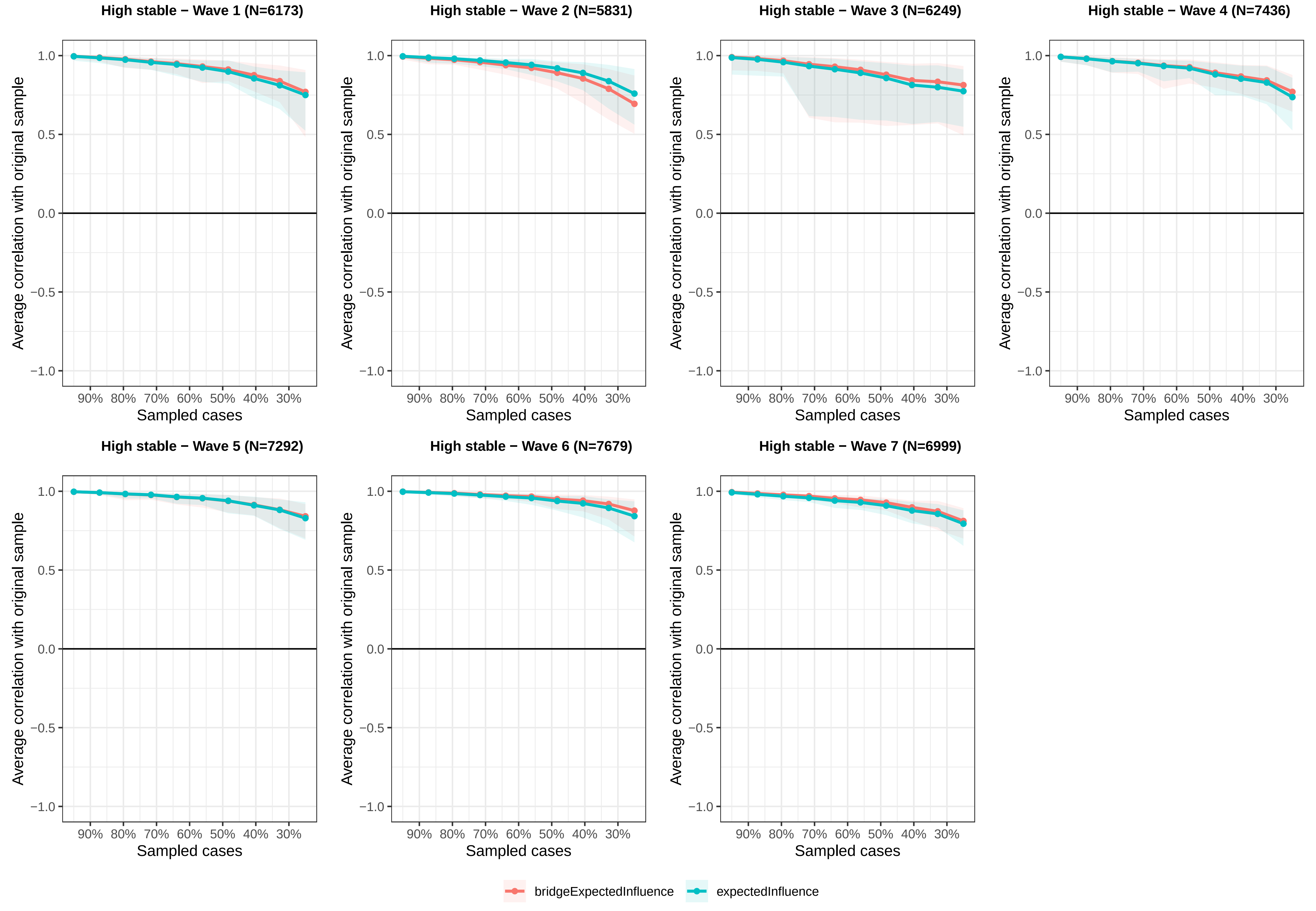
High stable − Wave 1 (N=6173)
High stable − Wave 2 (N=5831)
High stable − Wave 3 (N=6249)
High stable − Wave 4 (N=7436)
High stable − Wave 5 (N=7292)
High stable − Wave 6 (N=7679)
High stable − Wave 7 (N=6999)
Average correlation with original sample
Sampled cases
1.0
0.5
0.0
−0.5
−1.0
90% 80% 70% 60% 50% 40% 30%
bridgeExpectedInfluence
expectedInfluence
