# Supplementary material for: Are healthy ageing trajectories suitable to identify rehabilitation needs of the ageing population? An exploratory study using ATHLOS cohort data
Source: PLoS One. 2024 Jul 9;19(7):e0303865. doi: 10.1371/journal.pone.0303865 (PMC11232974; doi:10.1371/journal.pone.0303865)
Supplement: S11 Fig — The x-axis represents the edges’ weights, while every line on the y-axis represents a specific edge (not shown). The red line shows the estimate of the edge weights for each wave, and the grey bars the 95% confidence intervals of the bootstrap means of edge weights. (PDF) [file pone.0303865.s012.pdf]

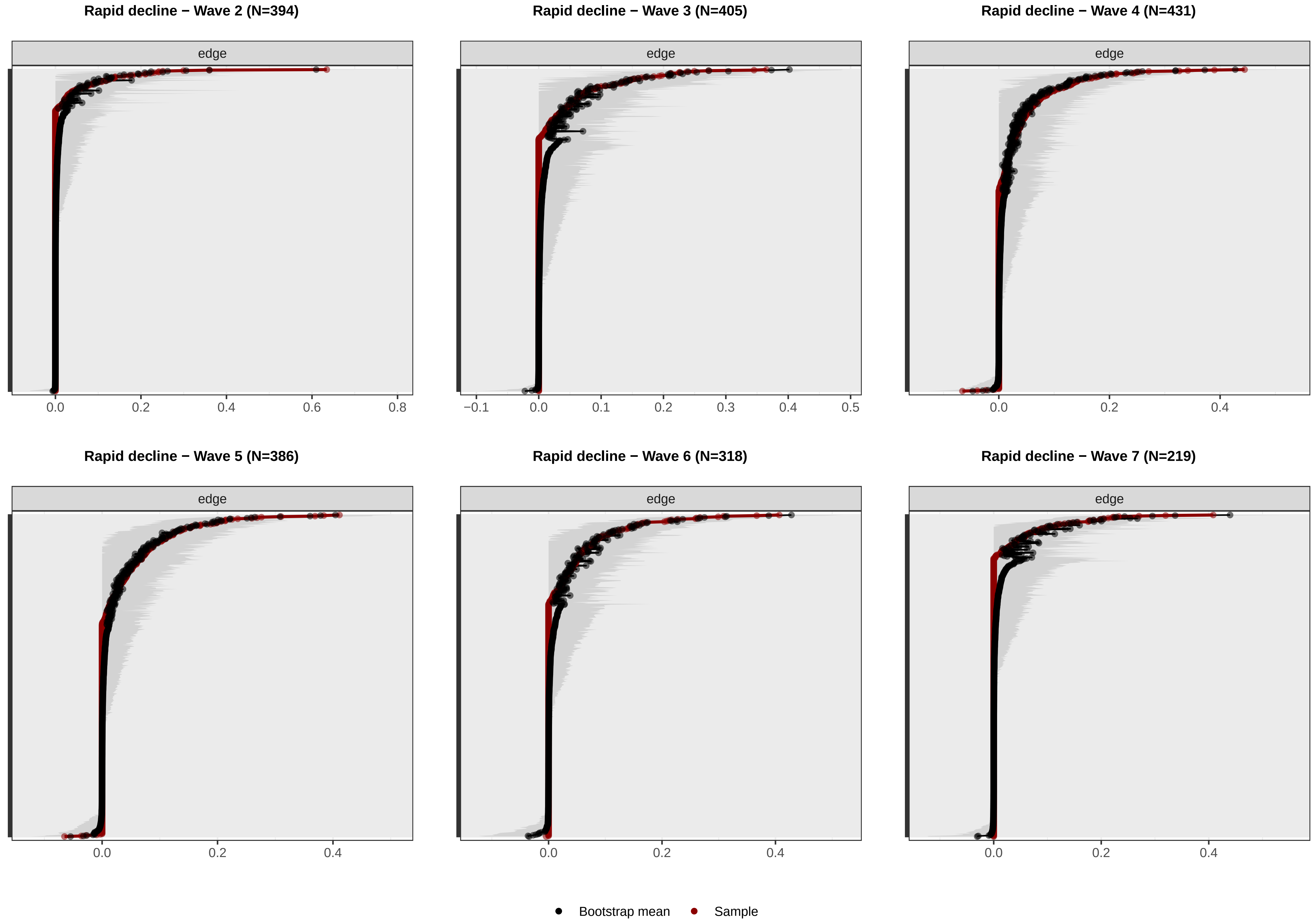
Rapid decline − Wave 2 (N=394)
edge
0.0
0.2
0.4
0.6
0.8
Rapid decline − Wave 3 (N=405)
edge
−0.1
0.0
0.1
0.2
0.3
0.4
0.5
Rapid decline − Wave 4 (N=431)
edge
0.0
0.2
0.4
Rapid decline − Wave 5 (N=386)
edge
0.0
0.2
0.4
Rapid decline − Wave 6 (N=318)
edge
0.0
0.2
0.4
Rapid decline − Wave 7 (N=219)
edge
0.0
0.2
0.4
Bootstrap mean
Sample
