# Supplementary material for: Are healthy ageing trajectories suitable to identify rehabilitation needs of the ageing population? An exploratory study using ATHLOS cohort data
Source: PLoS One. 2024 Jul 9;19(7):e0303865. doi: 10.1371/journal.pone.0303865 (PMC11232974; doi:10.1371/journal.pone.0303865)
Supplement: S12 Fig — The x-axis represents the edges’ weights, while every line on the y-axis represents a specific edge (not shown). The red line shows the estimate of the edge weights for each wave, and the grey bars the 95% confidence intervals of the bootstrap means of edge weights. (PDF) [file pone.0303865.s013.pdf]

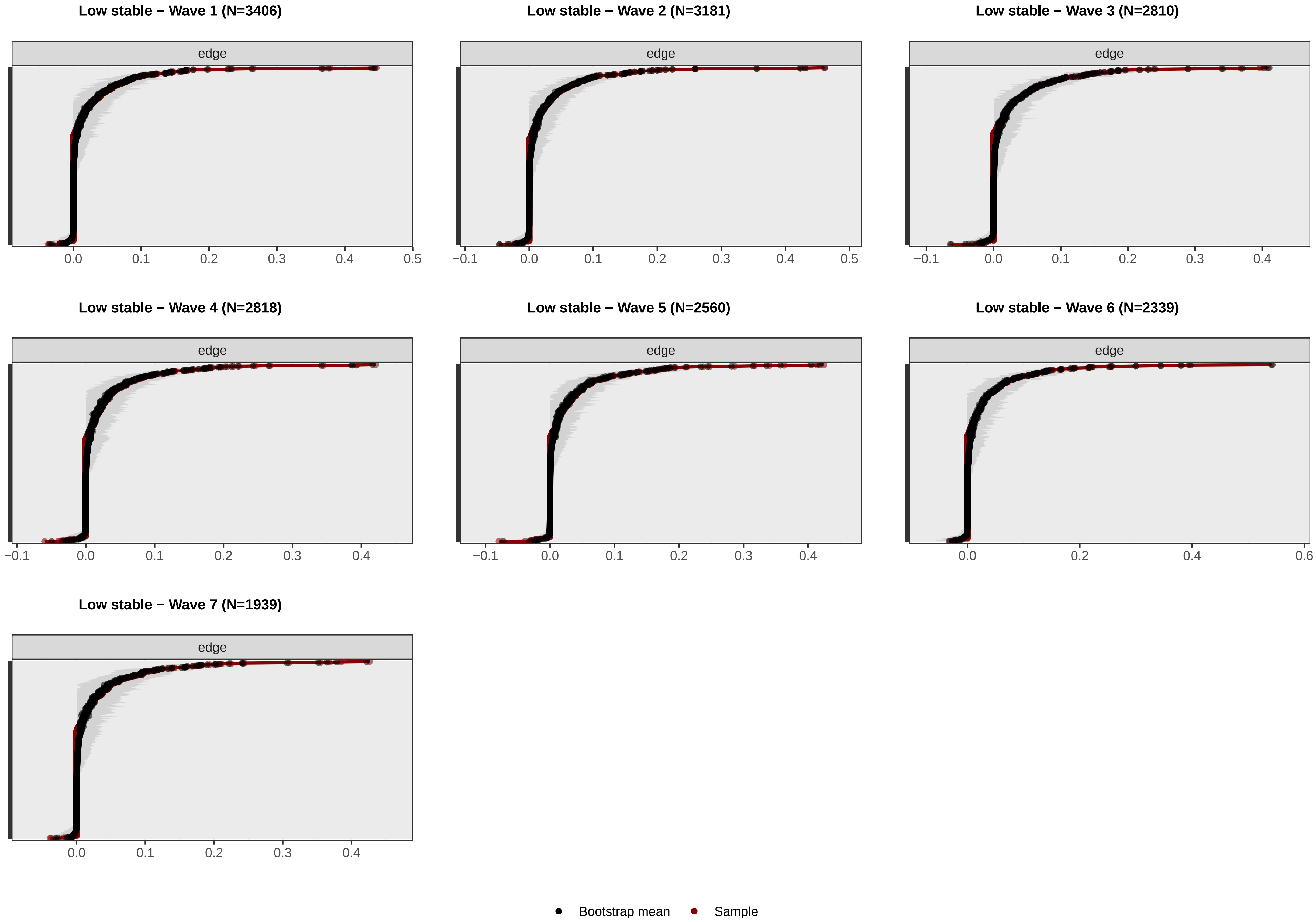
Low stable − Wave 1 (N=3406)
edge
0.0 0.1 0.2 0.3 0.4 0.5
Low stable − Wave 2 (N=3181)
edge
−0.1 0.0 0.1 0.2 0.3 0.4 0.5
Low stable − Wave 3 (N=2810)
edge
−0.1 0.0 0.1 0.2 0.3 0.4
Low stable − Wave 4 (N=2818)
edge
−0.1 0.0 0.1 0.2 0.3 0.4
Low stable − Wave 5 (N=2560)
edge
−0.1 0.0 0.1 0.2 0.3 0.4
Low stable − Wave 6 (N=2339)
edge
0.0 0.2 0.4 0.6
Low stable − Wave 7 (N=1939)
edge
0.0 0.1 0.2 0.3 0.4
Bootstrap mean
Sample
